# Phylogenetic reconstruction and species delimitation in Stipeae with special reference to *Stipa* (Poaceae, Pooideae) using mitochondrial genomes

**DOI:** 10.1111/cla.12618

**Published:** 2025-05-28

**Authors:** Katarzyna Krawczyk, Mateusz Maździarz, Łukasz Paukszto, Marcin Nobis, Jakub Sawicki

**Affiliations:** ^1^ Department of Botany and Evolutionary Ecology University of Warmia and Mazury in Olsztyn Plac Łódzki 1 Olsztyn warmińsko‐mazurskie 10‐727 Poland; ^2^ Faculty of Biology, Institute of Botany Jagiellonian University Gronostajowa 3A Kraków małopolskie 30‐387 Poland

## Abstract

Compared to plastid genomes, plant mitochondrial genomes have been less frequently used for species discrimination and phylogenetic studies due to assembly challenges, lower substitution rates and rapid structural evolution. However, this study demonstrates that mitochondrial genome fragments can be valuable for both molecular species identification and phylogenetic analysis in grasses of the tribe Stipeae. To explore this potential, we first assembled the complete mitochondrial genome of *Nassella tenuissima*—the first fully described mitogenome in Stipeae—which served as a reference for selecting 29 aligned mitochondrial genome fragments totalling 139 680 bp. These fragments were then analysed across 49 representatives of the tribe, including 43 *Stipa* species and six other taxa. The mitochondrial fragments achieved a species discrimination efficiency of 75%, slightly exceeding the 71% efficiency observed for plastid super‐barcodes. Additionally, comparative phylogenetic analyses using plastid and mitochondrial genomes underscored the utility of mitochondrial data in resolving phylogenetic relationships within Stipeae. Our findings provide a valuable resource for future research in transcriptomics, comparative genomics, phylogenomics and phylogeography of grasses.

## Introduction

Organellar genomes are widely used in plant evolutionary research, including phylogenetics, taxonomy, population genetics and phylogeography (Huang et al., 2019; Abdullah et al., 2021). The same characteristics that make them valuable for these applications also render them useful for molecular species identification—namely, their predominantly uniparental inheritance, relatively high level of polymorphism and presence in multiple copies per cell (Palmer, 1985; Henriquez et al., 2014; Ramsey and Mandel, 2019). So far, in plant research the use of plastid genomes has dominated due to their generally faster rate of evolution and, consequently, greater variability, as well as smaller size and simpler structure than mitochondrial genomes (Smith and Keeling, 2015). However, in recent years, mitogenomes have emerged as an alternative data source for plant phylogenetics, phylogeographic and ecological studies, complementing plastid genomes and nuclear sequences (Van de Paer et al., 2018; Huang et al., 2019; Szandar et al., 2022; Ke et al., 2023; Wang et al., 2024). Fortunately, advances in long‐read sequencing technologies have facilitated the assembly of these genomes, which are characterized by high structural variability and frequent genome rearrangements (Sloan et al., 2012). Understanding the variability of mitochondrial genomes sheds new light on plant evolutionary history, which is particularly valuable in the case of taxonomic groups in which frequent hybridization and introgression phenomena make phylogeny reconstruction difficult.

A taxonomic group posing particular challenges both for phylogenetic studies and molecular species identification is the tribe Stipeae, commonly referred to as feather grasses or needle grasses, belonging to Poaceae, Pooideae (Barkworth et al., 2008; Hamasha et al., 2012; Romaschenko et al., 2012; Cialdella et al., 2014; Tkach et al., 2021). Species within this group are predominantly perennial plants distributed worldwide in temperate and warm temperate regions, playing a crucial ecological role in forming grassland ecosystems, including steppes, semideserts and meadows (Barkworth and Torres, 2001; Nobis et al., 2020). Some species hold economic importance as forage plants, while others, as for example *Nassella tenuissima* (Trin.) Barkworth, are valued as ornamental species but also having invasive potential in Europe, Australia or New Zealand (Kaplan et al., 2025). Stipoid grasses are morphologically characterized by single‐flowered spikelets without a rachilla and lemmas with a sharp point or a terminal awn, which is entered by the keel and lateral veins (Cialdella et al., 2014). According to the latest classification of Poaceae, the tribe Stipeae includes more than 600 species classified into 34 genera (Soreng et al., 2022). The current scope and classification of the tribe have been shaped by recent research integrating classical taxonomy, based on morphological traits (Barkworth, 1993; Nobis et al., 2019, 2020) and phylogenetic studies (Romaschenko et al., 2010; Cialdella et al., 2014; Sclovich et al., 2015; Krawczyk et al., 2017; Peterson et al., 2019; Tkach et al., 2021; Krzempek et al., 2024). As a result of taxonomic revisions, the delimitation of some genera has changed significantly, including *Stipa*, the largest genus within the tribe, which now comprises over 150 species (Nobis et al., 2020). Several subgenera formerly recognized within *Stipa* have been elevated to generic rank, such as *Anatherostipa*, *Austrostipa*, *Jarava*, *Nassella* and *Pappostipa* (Cialdella et al., 2010; Romaschenko et al., 2008; Barkworth, 1990; Jacobs and Everett, 2000). Additionally, some species have recently been excluded from *Stipa* or *Achnatherum* and reassigned to genera such as *Stipellula*, *Barkworthia*, *Neotrinia*, *Pseudoeriocoma*, *Ptilagrostiella* and *Thorneochloa* (Hamasha et al., 2012; Peterson et al., 2019; Nobis et al., 2020; Tkach et al., 2021).

Recent studies have advanced our understanding of Stipeae evolution, estimating divergence times for Stipeae and *Stipa* (Baiakhmetov et al., 2021; Sha et al., 2023) and refining taxonomic relationships within the tribe and *Stipa* itself. However, some phylogenetic relationships remain unresolved and require further investigation. This is partly due to the fact that many species within feather grasses are poorly characterized, and a more comprehensive sampling of the group could improve phylogenetic reconstructions and assessments of interspecies diversity. Research on Stipeae is further complicated by the widespread distribution of its representatives, with some species exhibiting highly restricted ranges, including high‐elevation habitats (Barkworth and Torres, 2001; Nobis et al., 2020; Vintsek et al., 2022; Sultan et al., 2025) making it challenging to obtain plant material for comprehensive taxonomic studies. Additionally, hybridization events within *Stipa* (Nobis et al., 2019, 2020; Baiakhmetov et al., 2021; Sinaga et al., 2024) hinder taxonomic identifications.

However, the greatest challenge in reconstructing the evolutionary history of the *Stipa* genus and closely related Stipeae taxa is the low genetic variability of plastid sequences, which have been the primary data source in previous studies. Our prior research has demonstrated that even complete plastome analyses fail to generate highly reliable phylogenetic trees for this group. Moreover, the use of complete plastid genomes for species identification as so‐called super‐barcodes has shown limited effectiveness, differentiating only 56% of tested species within the genus, placing it among the most challenging groups to discriminate within Poales (Krawczyk et al., 2023).

Given the unexpectedly low variability of plastid genomes, we shifted our focus to identifying more effective genetic markers within the nuclear genome. Our studies led to the selection of the intergenic spacer region (IGS) within the nucleolar organizing region as a potential phylogenetic marker (Krawczyk et al., 2017). This marker exhibited superior phylogenetic informativeness compared to previously tested markers. However, the IGS region evolves rapidly, characterized by multiple repeat motifs, complicating sequence alignment and homologous site determination, which are essential for reliable phylogenetic inference. Additionally, as a biparentally inherited nuclear marker, IGS exhibits diversity between haplotypes, which must be considered in phylogenetic analyses. The need to find more effective genetic markers for studying Stipeae prompted us to search for them within the mitochondrial genome.

Referring to those findings, we aim to test whether phylogenetically informative regions can be identified within the mitochondrial genome of Stipeae and to define specific markers capable of effectively discriminating species within this group. For this purpose, the *N. tenuissima* mitogenome was thoroughly characterized, a species that nowadays is regarded as the most widespread Stipeae representative, including the identification of plastid‐derived sequences, codon usage analysis and comparison with the genome of *Stipa capillata*, as well as unpublished data on mitochondrial sequences of stipoid grasses. Both *N. tenuissima* and *S. capillata* are widely distributed taxa; however, *N. tenuissima* is in the Americas with numerous anthropogenic localities in Eurasia, whereas *S. capillata* is in Eurasia and north Africa (Cialdella et al., 2013; GBIF, 2025; Kaplan et al., 2025). Both of these species are also used as ornamental plants. The analysis of plastid‐derived sequences and repeated regions helped us to select fragments of the mitochondrial genome potentially useful in phylogenetic inference within the tribe of Stipeae. The comparison of the mitogenome‐based phylogenetic tree with a plastome‐derived one helped us to assess the phylogenetic informativeness of mitochondrial sequences.

## Materials and methods

### Ethics statement

Plant material collection complied with local and national regulations. For collection of species protected in Poland, official consent was given by Regional Directorates for Environmental Protection in Bydgoszcz and Gorzów Wielkopolski (Decisions No. WPN.6400.16.2013.JC.1, WPN.6400.26.2015.JC, WPN‐I‐6205.25.2015.AI, WPN‐I.6400.61.2014.AT.). Collection of plant material conducted in other countries did not require any permits. Plant samples were collected exclusively on public land.

### Taxon sampling and data acquisition

The research covered 33 species representing *Stipa*, *Achnatherum*, *Macrochloa*, *Nassella*, *Stipellula* and *Trikeraia*, all belonging to the tribe Stipeae. The pool of tested individuals was 50, of which data on complete plastid genomes were obtained for 48, while data on the tested mitochondrial fragments were analysed for 49 individuals (Table S1), including *N. tenuissima*, for which a complete mitochondrial genome was assembled and described. In the study, sequences of genomes described for the purposes of our previous work were used (Krawczyk et al., 2023), as well as newly sequenced data and records from GenBank. Plant names were accepted after Nobis et al. (2020).

### Sequencing, assembly and annotation

The libraries were prepared with short‐read sequencing (Illumina) or using nanopore‐sequencing technology (Oxford Nanopore Technologies) (Table S1). The details on library preparation, validation, quantification and sequencing of previously published genomes were described in detail in Myszczyński et al. (2016) and Krawczyk et al. (2018). Complete plastid genomes were assembled and annotated according to previously published pipelines (Myszczyński et al., 2016; Sawicki et al., 2017).

The mitochondrial genome of *N. tenuissima* was assembled *de novo* using Flye 2.9.3 software (Kolmogorov et al., 2019) and verified manually. Genes were annotated onto the complete genomes using the annotation transfer from *Lolium perenne* and *Zea mays* in Geneious Prime 2024 (Biomatters Ltd, Auckland, New Zealand). Analysis of the presence of structural variants of plastomes within analysed species was carried out using the Cp‐Hap pipeline (Wang et al., 2019).

Genome fragments for further analyses were selected manually, taking into account the criteria of the absence of MTPTs, the absence of repeated regions, and the commonness of these fragments in the species studied.

### The analysis of sequence variation

Sequences were aligned in Geneious Prime 2024, visually checked and trimmed. The number of variable and parsimony informative sites in the alignment was calculated in MEGAX (Kumar et al., 2018); the share of identical sites, pairwise identity and GC content were assessed using Geneious Prime 2024.0.3. The analysis of Molecular Diagnostic Characters (MDCs) utilized FastaChar v.0.2.4 software (Merckelbach and Borges, 2020). Each species was compared with other members of the same genus included in the study to generate a list of diagnostic features for individual species. FastaChar facilitates the examination of MDCs, which are defined as polymorphic sites (commonly SNPs) in nucleotide alignment. These sites exhibit a variant present in all members of the query taxon but not in any member of the reference taxa. Polymorphic sites within the query taxon were excluded from the analysis (Sarkar, 2008). The number of MDCs was additionally defined for two pairs of subspecies: *S. pennata* versus *S. pennata* subsp. *ceynowae*, *S. richteriana* vs *S. richteriana* subsp. *jagnobica* and also for *Achnatherum sibiricum* and *Macrochloa tenacissima*.

### Phylogenetic reconstruction

Phylogenetic inference was performed on the basis of complete plastid sequences, excluding one Inverted Repeat region, while in the case of the mitogenome, only concatenated alignments of unique 29 regions were used to construct the phylogenetic tree.

The phylogenetic trees constructed for plastid and mitochondrial sequences were built using the Maximum Likelihood (ML) method implemented in IQ‐TREE 2 (Minh et al., 2020) *N. tenuissima* and *N. trichotoma* sequences were selected as the root of the tree. The bootstrap consensus tree was inferred from 1000 replicates. The best‐fit model chosen with auto‐model selection according to BIC was K3Pu + F + I. Mitochondrial and plastid trees were compared using the cophylo function of the phytools 1.0‐3 R package (Revell, 2024).

### Comparative genomic analysis

To compare the structural homology of the *N. tenuissima* mitogenome with the previously described mitogenome of its close relative *S. capillata*, the sequences of these two species were aligned by the MashMap3 (Kille et al., 2023) tool implemented in SYNY software (Julian and Pombert, 2024). The potential gene transfers between the plastome and mitogenome of *N. tenuissima* have been revealed by the Blastn (Camacho et al., 2009) tools with cut‐offs: *e*‐value <10^−5^ and minimum alignment blast score >100.

### Relative synonymous codon usage analysis

Coding sequences from plastid and mitochondrial genomes were analysed using the RSCUcaller v.0.0.1 package (“GitHub—Mordziarz/RSCUcaller: Program in R for calculating RSCU”, 2024). For the analysis of RSCU in the mitogenome, the same alignment was used, on the basis of which the phylogenetic tree was calculated. Only the c18 fragment was removed because it did not occur in *A. sibiricum* and *S. capensis*. This resulted in the removal of the *nad*6 gene from the set of analysed data. In total, RSCU analysis was performed for 61 mitochondrial coding sequences. The significance of codon changes within a single amino acid was determined by the Kruskal–Wallis test. Changes within an amino acid were considered significant at a *P*‐value <0.05. Subsequently, variability among individual codons was determined by Dunn's *post hoc* test and Bonferroni correction. Differences were considered statistically significant when adjusted *P*‐value *P*_adj_ <0.05. Additionally, codon variability between plastid (PT) and mitochondrial (MT) genomes was determined in a similar manner. The Kruskal–Wallis test, Dunn's post hoc test and Bonferroni correction were again used, and differences were considered significant when *P*_adj_ <0.05. Pearson correlation was also employed to assess the concordance of codons in the PT and MT genomes of *N. tenuissima*. Pearson correlations were also determined between the summed codons of the PT and MT genomes. Additionally the MILC method with a hard filtering option to remove sequences shorter than 80 AA, as implemented in the coRdon 1.20.0 package, was applied to calculate the difference in codon usage distance between PT and MT PCGs (protein‐coding genes) (Supek and Vlahoviček, 2005).

### Visualization

The circle plots were generated using circos.ca (Krzywinski et al., 2009). The remaining graphics were produced in the R environment. Heatmaps were created using ComplexHeatmap v.2.18.0 (Gu et al., 2016). The phylogenetic tree was constructed with the phytools v.2.3.0 package (Revell, 2024). The rest of the figures were made using the ggplot2 package (“ggplot2: Elegant Graphics for Data Analysis (3e)”, 2016).

## Results

### Characteristics of the *Nasella tenuissima* mitogenome and assessment of its usefulness for phylogenetic inference in Stipeae

*Nassella tenuissima* is the first species in the tribe Stipeae with a complete mitochondrial genome described. Nanopore sequencing generated 5M reads containing in total 10.8 Gbp. *De novo* assembling resulted in a genome size of 424 370 bp with 400× coverage. Annotation of the mitogenome revealed the presence of 37 protein‐coding genes (PCGs), including all the mitochondrial core genes according to the classification of Skippington et al. (2015). A detailed description of the genome is given in a supplement (Appendix S1). The order and localization of genes are illustrated in Figs S1 and S2 and reported in Table S2. The results of relative synonymous codon usage are described in Appendix S1 and illustrated in Figs S3– S7.

**Fig. 1:**
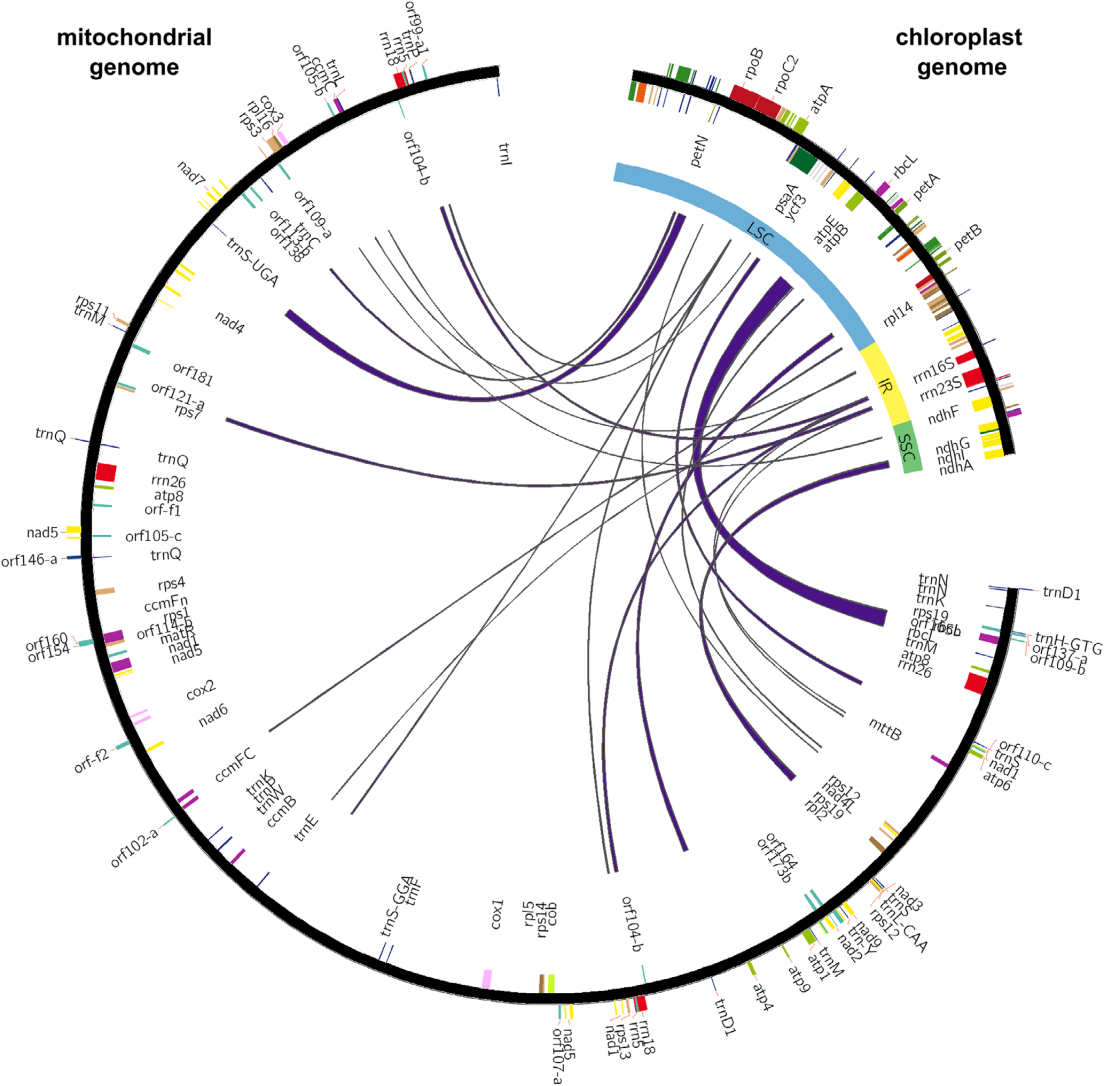
Shared linear mapping of mitochondrial and plastid homologous fragments in *Nassella tenuissima*. In the case of the plastid genome, only genes located near the transferred sequences have been described.

Within the mitochondrial genome, 30 regions of 64–4 310 bp length were identified as sequences highly homologous to plastid. It is possible that in the course of evolution they were transferred from the plastid to the mitochondrial genome, while retaining their copies in the plastid. As a result, only highly homologous genome fragments with blast scores equal to or exceeding 100 were considered. It should be noted that a fragment containing rrn16S in the plastome and rrn18S in the mitogenome was also identified as a homologous region, which is characterized by a relatively low identity (73.57%) and is most likely not a proof of transfer but only of sequence similarity of genes encoding analogous ribosomal subunits. The summary length of MTPTs is 19 293 bp, which accounts for 4.55% of the mitogenome. Their locations are characterized in Table S3 and shown in Fig. 1, where for better clarity, the figure shows the genome without one IR region. Altogether, 30 pairs of homologous regions between organellar genomes were described, some of which occurred in the mitogenome or in the plastome more than once. Excluding duplicates, there were 20 homologous regions observed, 17 of which included coding sequences, but mostly plastome‐derived regions contained only gene fragments that cannot be functional in the mitogenome. Five complete plastid genes are present in the mitogenome: *atp*E, *atp*B, *rbc*L, *ndh*I, *pet*N. The similarity of their sequences with respect to plastid homologues was 96.9%, 94.6%, 93.2%, 92.5% and 94.4%, respectively. Three of them, namely *atp*E, *atp*B and *rbc*L, lie within the longest of the identified homologous regions.

**Fig. 2:**
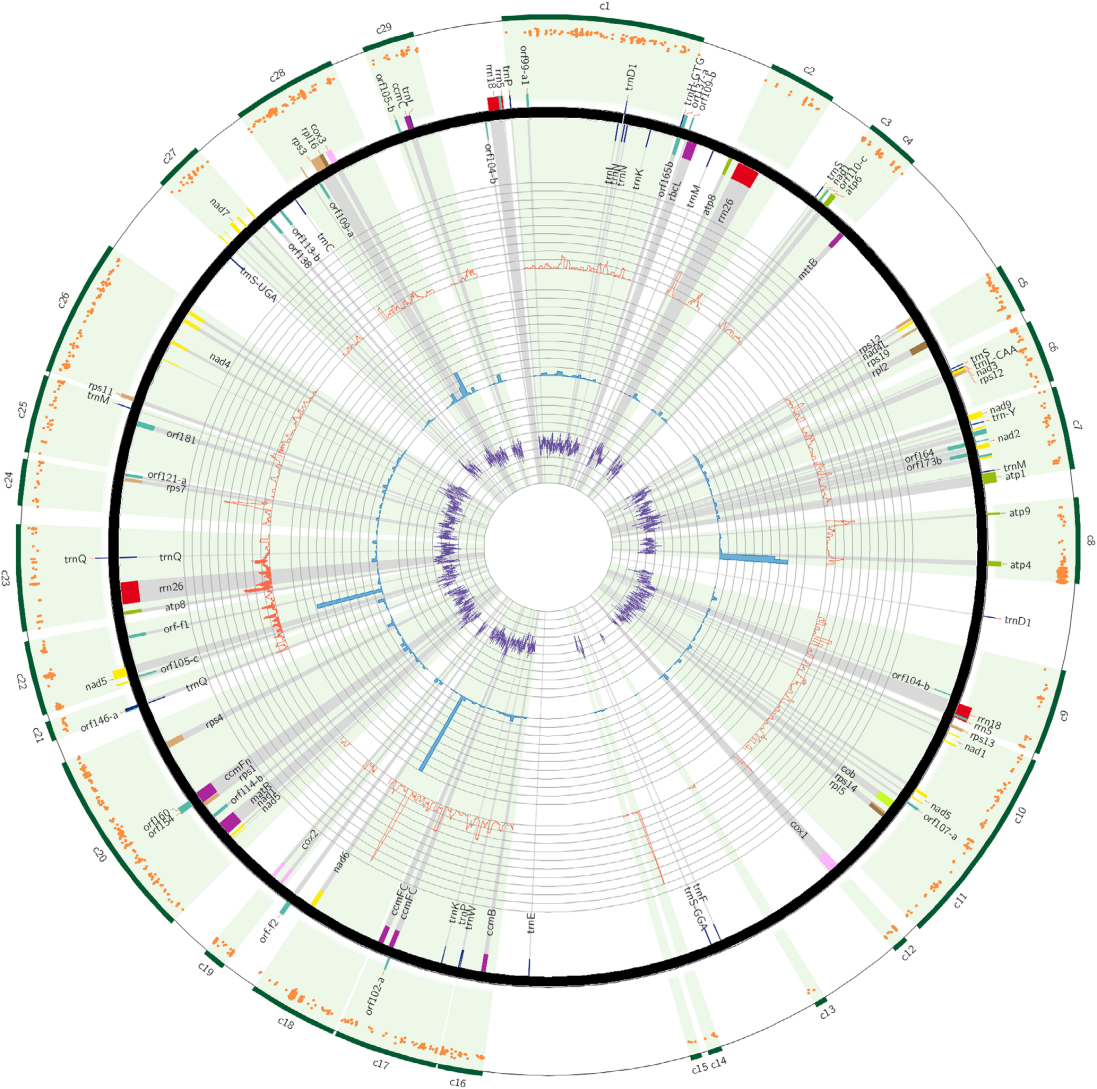
Characteristics of mitochondrial regions c1–c29 selected for studies on Stipeae. The outermost layer (green) shows the location of 29 selected genome fragments, which are additionally highlighted in light green; thick black line—the mitochondrial genome map of *Nassella tenuissima*; coloured bars around the black line depict coding regions, each of which is also represented by a grey highlight. Orange dots located on the outer axis of the circle correspond to individual MDCs. The first inner track (red line) shows pi diversity per 600 bp window size with a 100 bp shift. The next track (blue histogram) illustrates the number of MDCs in a 1000 bp window. The innermost track illustrates the G/C content in the *N. tenuissima* genome in a 100 bp window.

Within the mitochondrial genome, after excluding fragments present in the genome as a result of MTPT transfers, as well as repeated regions, 29 fragments of the mitogenome, common to the studied representatives of the Stipeae tribe, were selected as a set of data for further analyses. Their length after alignment ranged from 1 360 to 26 147 bp, and their exact location in the mitogenome, as well as their characteristics, are presented in Fig. 2 and Table S4. In general, the analysed fragments of the mitochondrial genome are characterized by low variability, as shown by very high pairwise identity values, exceeding in almost all cases 99%, and Pi diversity values equal to 0.0005. Among the analysed fragments, in terms of variability, the only fragment that stands out is c18, which was found to have a hypervariable fragment of 243 nt in length in *S. breviflora*, constituting 250 columns in the alignment.

Species‐specific mutation analysis shows that mitogenomes analysed individually demonstrate relatively low discriminatory power, as they allow the identification from 6.06% (c13) to 45.45% (c26) of the set of species analysed (Table S4; Fig. S8). However, the combined analysis of all 29 fragments of the mitogenome allows for the effective identification of 75.76% of the analysed species, and the use of complete plastome sequences as the so‐called super‐barcoding ensured identification success of 71%. The alignment length of mitogenome sequences was 266 654 bp, almost twice as long as the alignment length of plastid sequences, which amounted to 139 680 bp, and at the same time, the sum of MDCs in mitochondrial sequences (2786) was much lower than in the case of plastid sequences, where the sum of MDCs for all analysed species was 4 262. A comparison of species‐specific mutations observed in organelle genomes shows that despite the much smaller number of MDCs in mitochondrial sequences, both in absolute terms and in terms of sequence length, the species discrimination power is slightly stronger in the mitochondrial genome (Table S4). Among the analysed species, six had no species‐specific mutations neither in PT nor in MT data sets, that is: *S*. *× heptapotamica* and its parental species *S. lessingiana* in addition to *S. arabica*, *S. borysthenica*, *S. caucasica* and *S. zalesskii*.

Overall, a slower rate of accumulation of species‐specific substitutions was observed in the mitogenome than in the plastome. In turn, in the case of several species, the opposite tendency was observed. Moreover, an advantage in the accumulation of substitutions in MT over PT sequences was observed in: *S. purpurea* (224 vs 25), *S.× tzveleviana* (79 vs 2), *S. przewalskyi* (43 vs 34) and *S. austroaltaica* (10 vs 1).

In addition to the analysis of MDCs distinguishing species from the analysed set of species, a comparison of the number of MDCs for selected pairs of taxa was also performed. This analysis revealed that MT sequences were more effective in identifying *S. richterina* subsp. *richteriana* from *S. richteriana* subsp. *jagnobica* (35 vs 5) as well as *Achnatherum sibiricum* from *Stipellula capensis* (1 981 vs 1 474).

The number of variable nucleotides in mitochondrial sequences between *A. sibiricum* and *S. capensis* was comparable to that observed between *Macrochloa tenacissima* and *Trikeraia pappiformis* (2579) and much more significant than between *N. tenuissima* and *N. trichotoma* (67).

### Characteristics of plastid genomes

**Fig. 3:**
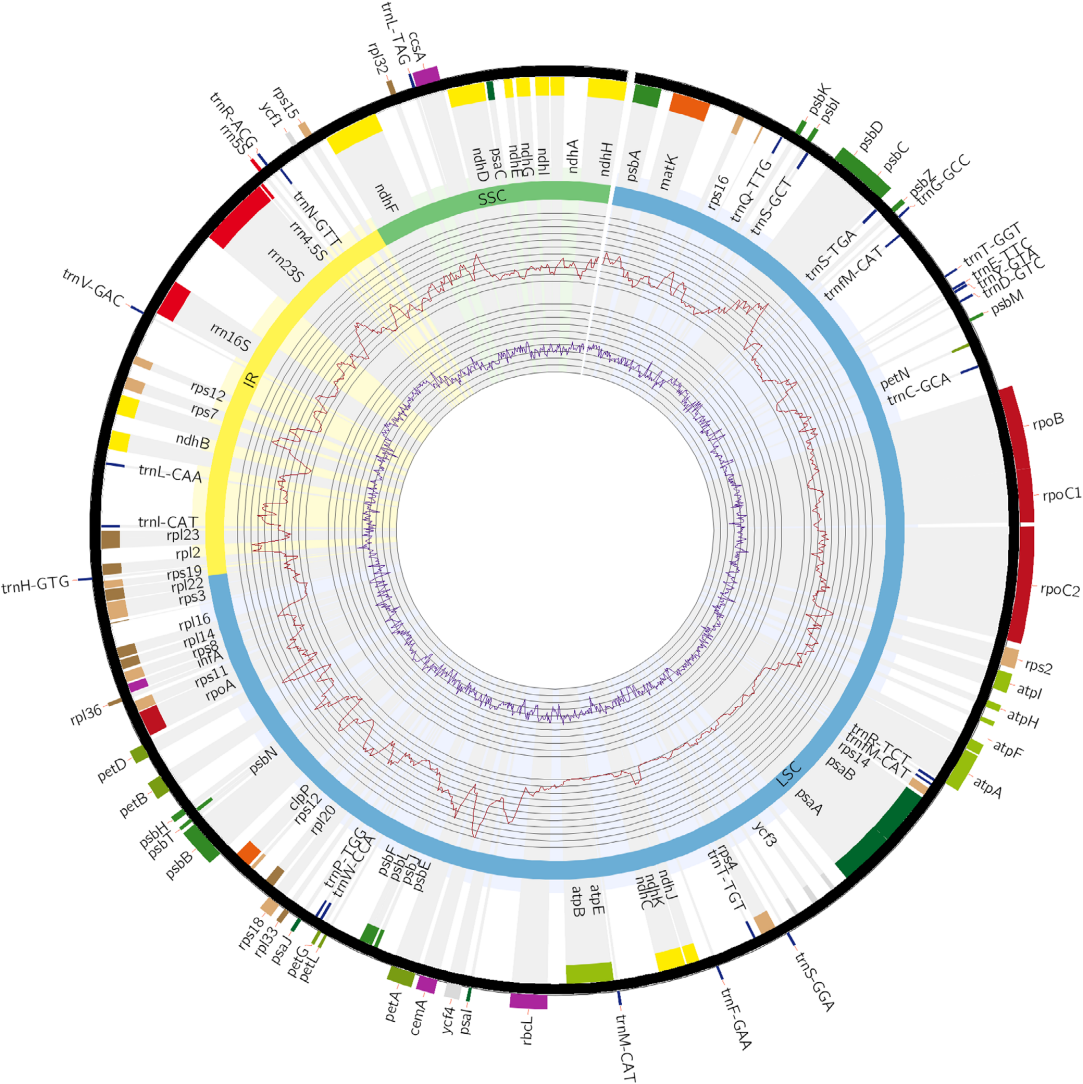
Plastome variability in Stipeae. The outermost track displays plastid genes with corresponding labels. Each gene is highlighted with a grey feature extending towards the centre of the circle. The first inner track characterizes the IR (yellow), SSC (green) and LSC (blue) regions, which are also marked with lighter features. The next track, marked with a red line, represents pi diversity in a 600 bp window. The final track, highlighted with a purple line, shows G/C content in a 100 bp window.

For the purposes of this study, the plastid genomes of two species were assembled: *Stipa tianschanica* and *N. tenuissima*. The organization of their genomes, as well as the composition and order of genes, was found to be consistent with those previously published for Stipeae (Fig. 3) (Myszczyński et al., 2016; Krawczyk et al., 2018), which proves the structural conservativeness of plastid genomes in this taxonomic group. A total of 48 plastid sequences was used in the study. The alignment of complete genome sequences had a length of 139 680 bp; however, for further analyses, the IR region was excluded, and the data set was 117 987 bp long with sequences ranging from 115 565 to 116 230 bp. It was characterized by a 37.8% GC content, 99.2% pairwise identity, Pi diversity mean equal to 0.0028, only 3 766 variable sites and 1 770 parsimony informative sites.

### Phylogenetic inference from organellar genomes

**Fig. 4:**
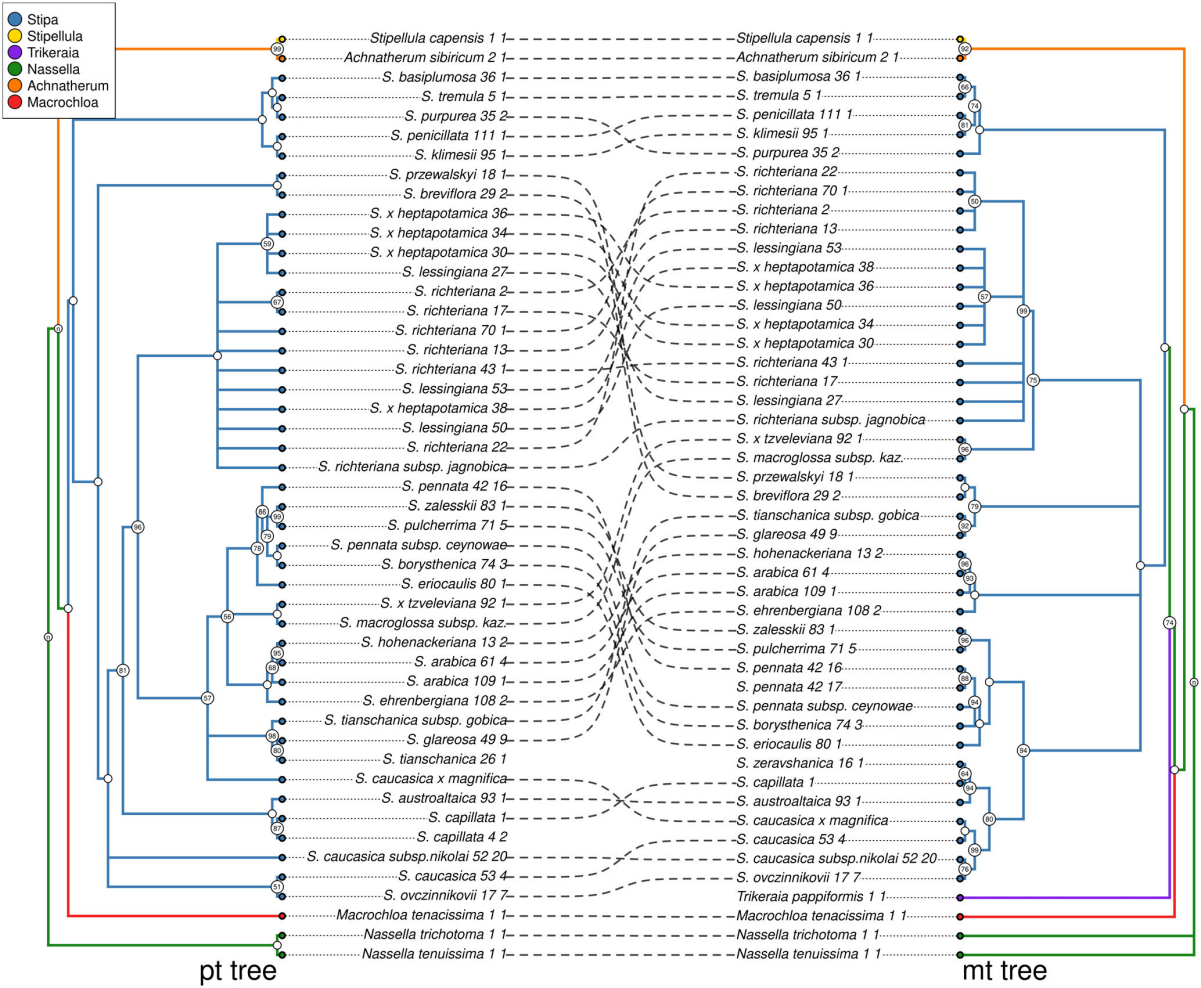
Comparison of ML trees based on plastid and mitochondrial data sets. Bootstrap values are given in circles at nodes. Nodes without a reported value have full (100%) bootstrap support.

The phylogenetic trees of the studied representatives of Stipeae obtained on the basis of data from the plastid genome and fragments of the mitochondrial genome are largely consistent with each other, although there are also some discrepancies between them (Fig. 4, Figs S9 and S10). In both cases, the trees are not completely resolved and contain polytomies, in the PT tree in five clades and in the MT tree in six clades. The similar overall level of credibility of the tree proves the comparable informativeness of data from both organelle genomes. There are even significantly fewer clades in the MT tree with bootstrap values below 60%, and these only concern the group including *S. lessingiana*, *S. richteriana* and their hybrid *S. × heptapotamica*. We can therefore risk saying that the PT tree is only apparently better developed, and for some low‐confidence clades, we observe cases of incongruence between the trees.

In the case of *S. ovczinnikovii* and *S. caucasica*, the MT data indicated a close relationship between these species, grouping all their representatives into a highly supported clade (98.65%) although within this the samples of S. caucasica form a paraphyletic group relative to *S. ovczinnikovii*. Therefore, in the case of *S. caucasica* and *S. caucasica* subsp. *nikolai*, the phylogenetic signal obtained from the MT data confirmed the former classification based on morphology.

Comparison of the results of phylogenetic reconstruction based on both organellar genomes allowed us to confirm the clear distinctiveness of species from the Himalayan group (*S. basiplumosa*, *S. klimesii*, *S. penicillata*, *S. purpurea*, *S. tremula*). However, the phylogenetic signal coming from the mitogenome additionally emphasizes the distinctiveness of the Central Asian group (*S. breviflora*, *S. glareosa*, *S. przewalskyi*, *S. tianschanica* subsp. *gobica*).

In both trees, the phylogenetic line leading to closely related *Achnatherum sibiricum* and *Stipellula capensis* separated from *Stipa* earlier than *Macrochloa* and *Trikeraia*. Also, both data sets indicate the monophyletic nature of the *Stipa* genus (BS = 100%) and distinguish a basal clade in it, which in both cases is formed by mountain species: *S. klimesii*, *S. penicillata*, *S. basiplumosa*, *S. purpurea* and *S. tremula*. The phylogenetic signal from both organelle genomes shows that *S. arabica* is closely related to *S. honenackeriana*, and *S. arabica* is a paraphyletic taxon.

There are also cases of conflicts in topology despite the high reliability of the clades of both trees. Thus, phylogenetic inference from MT data suggested that *S. austroaltaica* and *S. capillata* are a sister group to *S. caucasica*, while the PT data indicate an earlier lineage separation leading to *S. caucasica*. In turn, in the MT tree, the clade connecting *S. zalesskii* and *S. pulcherrima* is sister to the clade comprising *S. eriocaulis*, *S. pennata* and *S. borysthenica*, with very high credibility. Whereas, the PT tree indicated a close relationship between *S. zalesskii* and *S. pulcherrima* with *S. pennata* and *S. borysthenica* (Fig. 4), located in a common clade with *S. eriocaulis* as a sister to those above mentioned.

However, the obtained trees differ in the location of *S. purpurea*, which in the MT tree is located more internally in the discussed clade. What draws attention is the exceptionally long branch leading to this species, the presence of which indicates a large number of changes in the sequence in relation to the neighbouring species on the tree.

The second example of a very long branch and at the same time conflicting phylogenetic signal in relation to the PT data is the location in the trees of the clade connecting *S. breviflora* and *S. przewalskyi*, which in the PT tree is the second phylogenetic line separated from the genus after the Himalayan clade, while in the MT tree they constitute a clade sister to *S. glareosa* and *S. tianschanica* subsp. *gobica*.

## Discussion

*Nassella tenuissima*, next to *S. capillata*, is the second species in the Stipeae tribe for which the structure of the mitochondrial genome has been studied and the first which thanks to the use of nanopore long reads was assembled into a single circular chromosome. The circular model of plant mitochondrial DNA structure, previously considered to be common, turned out to be only one of the possible options, next to mitogenomes in the form of a loop, lasso or linear multi‐chromosomal genome structures (Sloan et al., 2012). With this knowledge, we carefully assembled the genome to ensure that the circular chromosome accurately represented its structure. So far, in the Poaceae family, exclusively circular mitochondrial genomes have been described, with *S. capillata* being the one that has been published in the form of four contigs (Baiakhmetov et al., 2021).

With a size of 424 370 bp, the *N. tenuissima* mitogenome is one of the smaller among the closely related species of the subfamily Pooideae. Contigs of *S. capillata* had a total length of 3.2% greater, in *Hordeum vulgare* by 23.9% (Hisano et al., 2016), in *Elymus magellanicus* by 37.5% (Chen et al., 2023) and in *Lolium* perenne by 59.9% more (Islam et al., 2013). In turn, the mitogenome of *N. tenuissima* is larger by 6.4% than that of *Aegilops longissima*, which is 399 005 bp long (Noyszewski et al., 2014). GC content observed in *N. tenuissima* accounting for 44.4% is at a very similar level to that of *L. perenne* (44.1%), *Avena longiglumis* (44.05%) and *H. vulgare* (44.2%) (Hisano et al., 2016; Liu et al., 2023). The mitogenome of *N. tenuissima* is characterized by almost the same gene content as in *S. capillata*; the differences only concern the number of copies of some genes. In *N. tenuissima*, the *atp*8 and *rps*12 genes occur in two copies, while in *S. capillata* one copy of each of these genes has been annotated (Baiakhmetov et al., 2021). In turn, in *S. capillata* there are two copies of *atp*6, while in *N. tenuissima* it occurs in one copy. Greater differences are observed in the numbers of orfs, as 29 have been identified in *N. tenuissima*, while 34 orfs have been annotated in *S. capillata*. Compared to species from the closely related tribe Triticeae, differences in gene composition are visible, such as the lack of *rps*2 in relation to *Hordeum vulgare* (Hisano et al., 2016) and the lack of *atp*H, *rpl*14, *rps*16 and *rps*8 reported for *Elymus magellanicus*. In turn, in relation to *Avena longiglumis* from the Poeae tribe, the differences concern the lack of the *sdh*4 gene (Liu et al., 2023), while in *L. perenne* there are differences in the genes encoding ribosomal proteins (Islam et al., 2013), which confirms the high variability of mitochondrial genomes in terms of PCG content even in close phylogenetic lines within Poaceae.

Grass mitogenomes known so far differ greatly also in terms of plastome‐derived regions. Their share in the genome itself ranges in Poaceae from 1.5% in *A. longiglumis* (Liu et al., 2023) to 7.11% in *Sorghum bicolor* (Xiong et al., 2022), with the score for *N. tenuissima* being 4.55%, placing it almost in the middle of this range. The comparison of PCGs transferred in MTPTs shows even more significant variation in plastome‐derived sequences between the species, reporting that MTPTs in the species from the same family have only single genes in common. A comparative analysis of organellar transfers, conducted on nine grass species representing seven genera: *Elymus*, *Triticum*, *Saccharum*, *Zea*, *Oryza*, *Sorghum*, *Aegilops* and *Eleusine* (Xiong et al., 2022), revealed that almost all the transferred genes of the mitogenomes coded for tRNA and rRNA. From protein‐coding sequences, the *atp*4 gene was transferred between the mitochondrial and plastid genomes of *Zea perennis*. In turn, the result of our study on *N. tenuissima* showed that the *atp*4 gene was not transferred to the mitogenome, either wholly or in part. A comparison of the results obtained for *H. vulgare* (Hisano et al., 2016) and *N. tenuissima* mitogenomes shows that only a fragment of the *psa*A gene was transferred in both of them. The largest number of common plastome‐derived PCGs is observed between the *N. tenuissima* mitogenome and *A. longiglumis* from Poeae (Liu et al., 2023), where three (out of 10) partially transferred genes, namely: *atp*A, *ndh*A and *rpl*14 overlapped with the transfers observed in *N. tenuissima*. Such large differences in the share of MTPTs in grasses suggest that the sequence transfer phenomenon between organelles could not have been a one‐time event, as hypothesized by Palmer (1985).

Knowledge of the plastid‐mitochondrial transfer phenomenon is important not only for comparative genomics and potential evolutionary and physiological inferences but also from the point of view of molecular species identification; therefore, we excluded sequences present in both organellar genomes from the further analysed data set. Due to the significant share of MTPTs in the mitogenome, including sequences used as DNA barcodes, the so‐called “barcoding paradox” may occur (Park et al., 2020), that is falsification of barcoding analysis due to amplification of both the plastid sequence and the one transferred to the mitogenome. In the case of *N. tenuissima*, the *rbc*L gene, one of the most commonly used, classic plant DNA barcodes, was transferred, becoming a sequence present in both organelle genomes.

Unlike plastid sequences, the mitochondrial genome is not commonly used in plant species identification. This is primarily due to the slower evolution rate of mtDNA coding sequences, which in seed plants is 3–4 times lower than in the case of ptDNA sequences (Drouin et al., 2008; Richardson et al., 2013). Such a low mutation rate is probably the result of efficient DNA repair mechanisms, in particular repair by homologous recombination (Chevigny et al., 2020). Comparison of the overall variability of plastid genomes and selected fragments of the mitogenome in Stipeae confirmed lower variability of the mitogenome expressed. Despite this, the level of interspecific variation, as measured by the number of species‐specific substitutions, does not differ much between the plastome and mitogenome. Moreover, in some cases, where ptDNA failed, mtDNA sequences highlighted species differences. Perhaps the obtained result does not indicate high variability of the mitogenome, but rather confirms the extremely low variability of the plastome in *Stipa*, which has already been shown by our previous study comparing the level of this variability compared to other representatives of the Poales order (Krawczyk et al., 2023). The low effectiveness of species discrimination in Stipeae based on organellar genomes is probably largely influenced by hybridization and introgression events (Nobis et al., 2019; Sinaga et al., 2024) and probably also incomplete lineage sorting. According to our best knowledge, there is no literature data on flowering plants that would allow us to determine whether the observed level of mitogenome variability in Stipeae is high or low compared to the broader taxonomic group.

The obtained data confirm the applicability of the analysed mitochondrial sequences in the phylogenetic reconstruction of the Stipeae tribe and even in the case of many closely related species from the *Stipa* genus itself. It may also be useful in studies on other representatives of the Poaceae family.

The phylogenetic tree obtained on the basis of the mitogenome partially differs from that obtained by analysing the variability of the plastome, but these differences do not concern internal nodes and are consistent with respect to the mutual relationships of the studied representatives of the genera *Nassella*, *Macrochloa*, *Achnatherum* and *Stipellula*. Regarding the genus *Achnatherum*, however, it is necessary to extend the analyses to other species because literature data suggest a polyphyletic origin of this genus (Cialdella et al., 2010; Romaschenko et al., 2010).

The use of mitochondrial sequence variability in phylogenetic inference allowed us to obtain a stronger signal in the group of *S. caucasica* (including *S. caucasica* s.str. and *S. caucasica* subsp. *nikolai*), *S. capillata* (including *S. capillata* and *S. austroaltaica*), *S. purpurea* (including *S. purpurea* and *S. tremula*) and *S. pulcherrima* (including *S. pulcherrima* and *S. eriocaulis*) where the analysis of plastid sequences did not allow for unambiguous determination of relationships between these species. We also noted a specific discrepancy between ptDNA and mtDNA in the above‐mentioned groups of species. It is probably a result of the differences in evolution rate within and between the PT and MT. Nevertheless, it can also provide additional support for evolutionary diversification within these groups of taxa (Krawczyk et al., 2023; Sinaga et al., 2024). The observed discrepancy between phylogenetic signals obtained on the basis of plastomes and mitogenomes shows it to be not uncommon in Poales (Wu et al., 2022) and, similarly to the low efficiency of species identification, it may result from the complicated evolutionary history of the studied group, such as rapid radiation, hybridization, the formation of hybrid swarms, incomplete lineage sorting (Wu et al., 2022) or even horizontal genome transfer (Hertle et al., 2021).

In turn, if the inconsistency between trees is associated with the presence of a particularly long branch in the MT tree, as in the case of *S. purpurea* and *S. breviflora*, it can be assumed that it results rather not from the history of this species but from the specificity of the maximum likelihood method, which is sensitive to the effect of attracting long branches. The presence of such long branches, indicating high genetic variability between closely related species, shows how dynamic the evolution of the mitochondrial genome can be. While abrupt changes or an increase in the rate of evolution in the lineages can be very useful in species discrimination, they can make phylogenetic reconstruction difficult.

## Conclusions

Comparative analysis of mitochondrial genomes within Stipeae, with a particular focus on *Stipa*, enabled us to identify mitochondrial genome fragments valuable for phylogenetic analysis within the Stipeae tribe. The phylogenetic signal inferred from the mitogenomes turned out to be not completely consistent with the signal generated by plastid data; however, it provides new, valuable information that helps in resolving the relationships between the studied species. The phylogenetic reconstruction of relationships within the genus *Stipa* remains an open issue as we still have only a fragmentary picture of this species‐rich and taxonomically challenging genus. A more complete picture of the phylogeny would probably be obtained by the analysis of nuclear genome variability based on multi‐locus analysis, which we consider a future prospect.

Moreover, the analysis of the variability of selected mitogenome fragments showed that despite a lower level of variability, their usefulness in species discrimination in *Stipa* is at a similar level to plastid genomes.

Our findings on mitogenome variability in Stipeae could lay the foundation for further research in transcriptomics, comparative genomics, phylogenomics and phylogeography of grasses.

## Funding

The study was supported by the National Science Centre, Poland (project no. 2023/51/B/NZ8/01179).

## Conflict of interest

The authors declare no competing interests.

## Author contributions

KK and JS designed the research; KK, JS, MM, ŁP, MN performed the research and analysed data, MN collected and verified plant material; MM and ŁP visualized the results; KK, MM and MN wrote the paper; all the authors verified and approved the final version of the manuscript.

## Supporting information


**Fig. S1.** Gene map of the *Nassela tenuissima* mitochondrial genome.

**Fig. S2.** Comparison of four mitochondrial contigs of *Stipa capillata* (left) and *Nassella tenuissima* (right) mitochondrial genome structure and gene order.

**Fig. S3.** Codon usage in mitochondrial and plastid genome of *Nassella tenuissima*.

**Fig. S4.** Statistically significant differences in codon usage frequencies between the plastomes and mitogenomes in Stipeae.

**Fig. S5.** The RSCU heatmap of mitochondrial codons shows species preference of codon usage: dark orange and light orange colours represent less preferred codons, white colour represents codons that are neither less preferred nor more preferred, and light blue and dark blue colours represent more preferred codons.

**Fig. S6.** The RSCU heatmap of plastome codons shows species preference of codon usage: dark orange and light orange colours represent less preferred codons, white colour represents codons that are neither less preferred nor more preferred, and light blue and dark blue colours represent more preferred codons.

**Fig. S7.** MILC analysis of codon usage bias between MT and PT genomes.

**Fig. S8.** Comparison of the effectiveness of species identification using the analysed mitobarcodes (left plot) and using plastomes (excluding one IR) and 29 mitobarcodes (right plot).

**Fig. S9.** Evolutionary analysis based on mitochondrial fragments (c1–c29 described in the text).

**Fig. S10.** Plastome‐based evolutionary analysis by Maximum Likelihood method based on the K3Pu + F + I model of nucleotide substitution.

**Table S1.** Specimens used in the study, source of sequences, GenBank accession numbers.

**Table S2.** Annotation of *Nassella tenuissima* mitogenome.

**Table S3.** Characteristics of potentially plastome‐derived mitochondrial sequences.

**Table S4.** Comparison of plastid genome and mitogenome fragments in terms of their effectiveness in species identification.

**Table S5.** Codon usage in *Nassella tenuissima* mitogenome and plastome.

**Appendix S1.** Characteristics of the mitochondrial genome of *Nassella tenuissima*.

## Data Availability

The data that supports the findings of this study are available in the supplementary material of this article.
