# Supplementary figures and images for: Phylogenetic reconstruction and species delimitation in Stipeae with special reference to Stipa (Poaceae, Pooideae) using mitochondrial genomes
Source: Cladistics. 2025 May 28;41(4):358–71. doi: 10.1111/cla.12618 (PMC12267929; doi:10.1111/cla.12618)

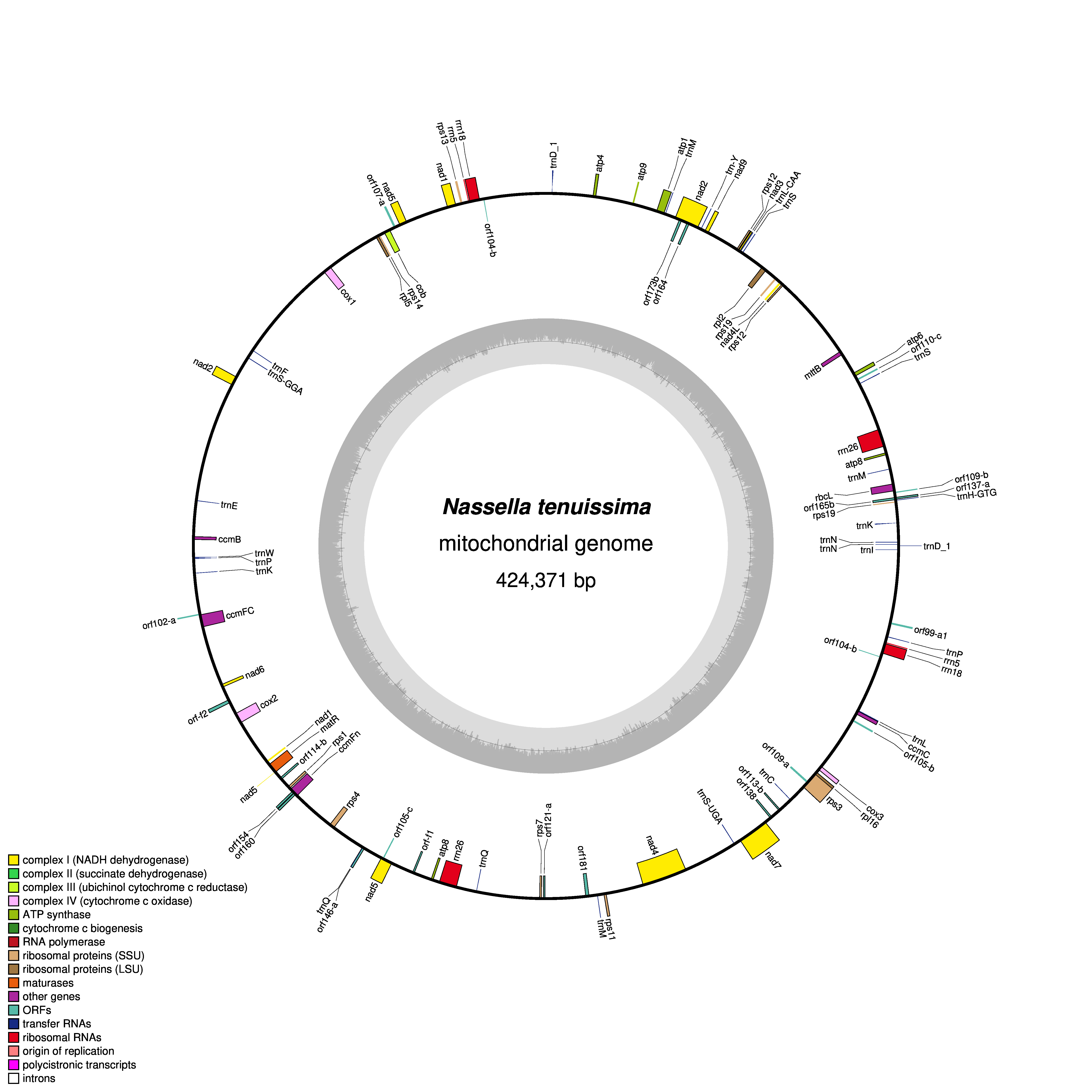

Supplement: Supplementary file 1 — Fig. S1. Gene map of the Nassela tenuissima mitochondrial genome. [file CLA-41-358-s003.jpg]

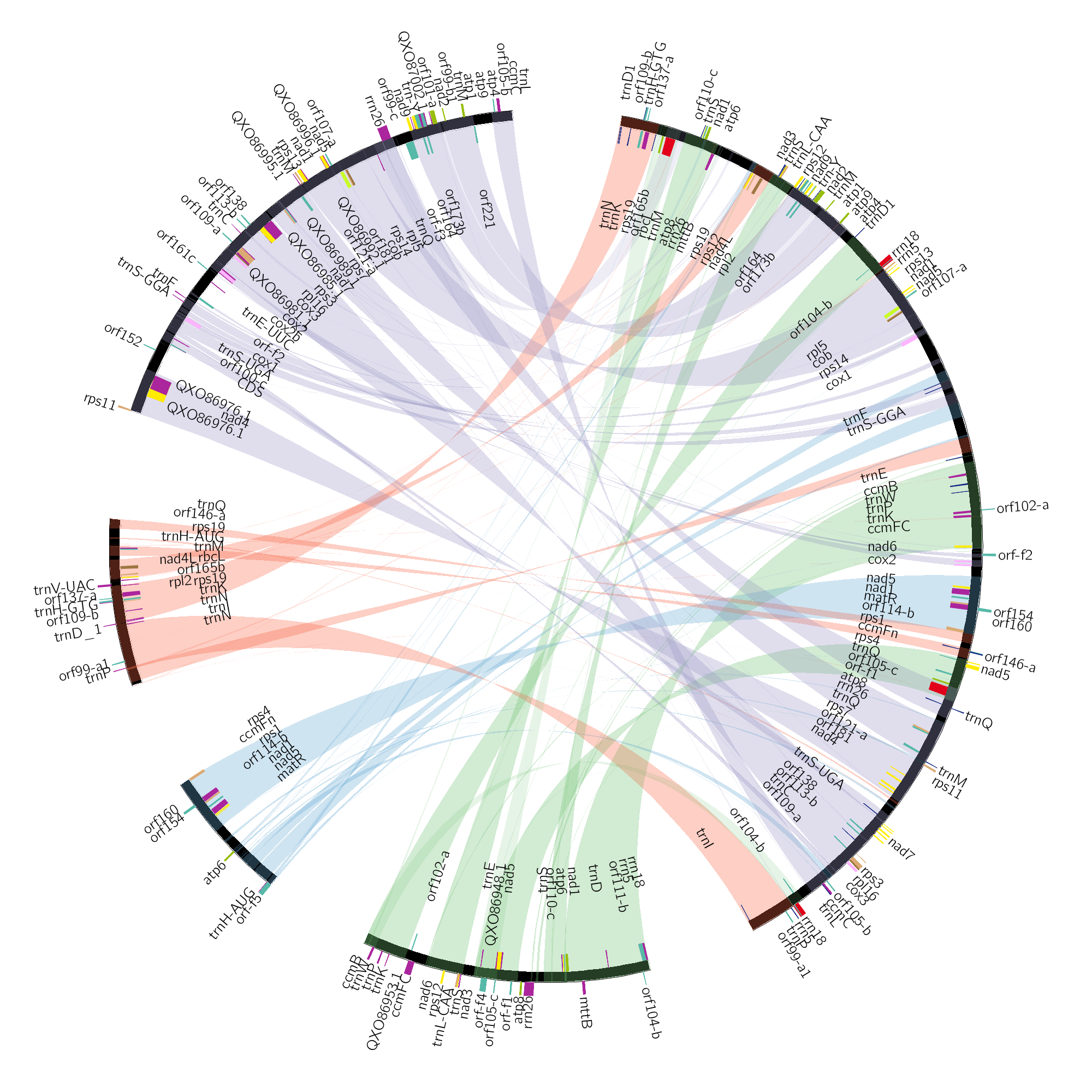

Supplement: Supplementary file 2 — Fig. S2. Comparison of four mitochondrial contigs of Stipa capillata (left) and Nassella tenuissima (right) mitochondrial genome structure and gene order. [file CLA-41-358-s010.png]

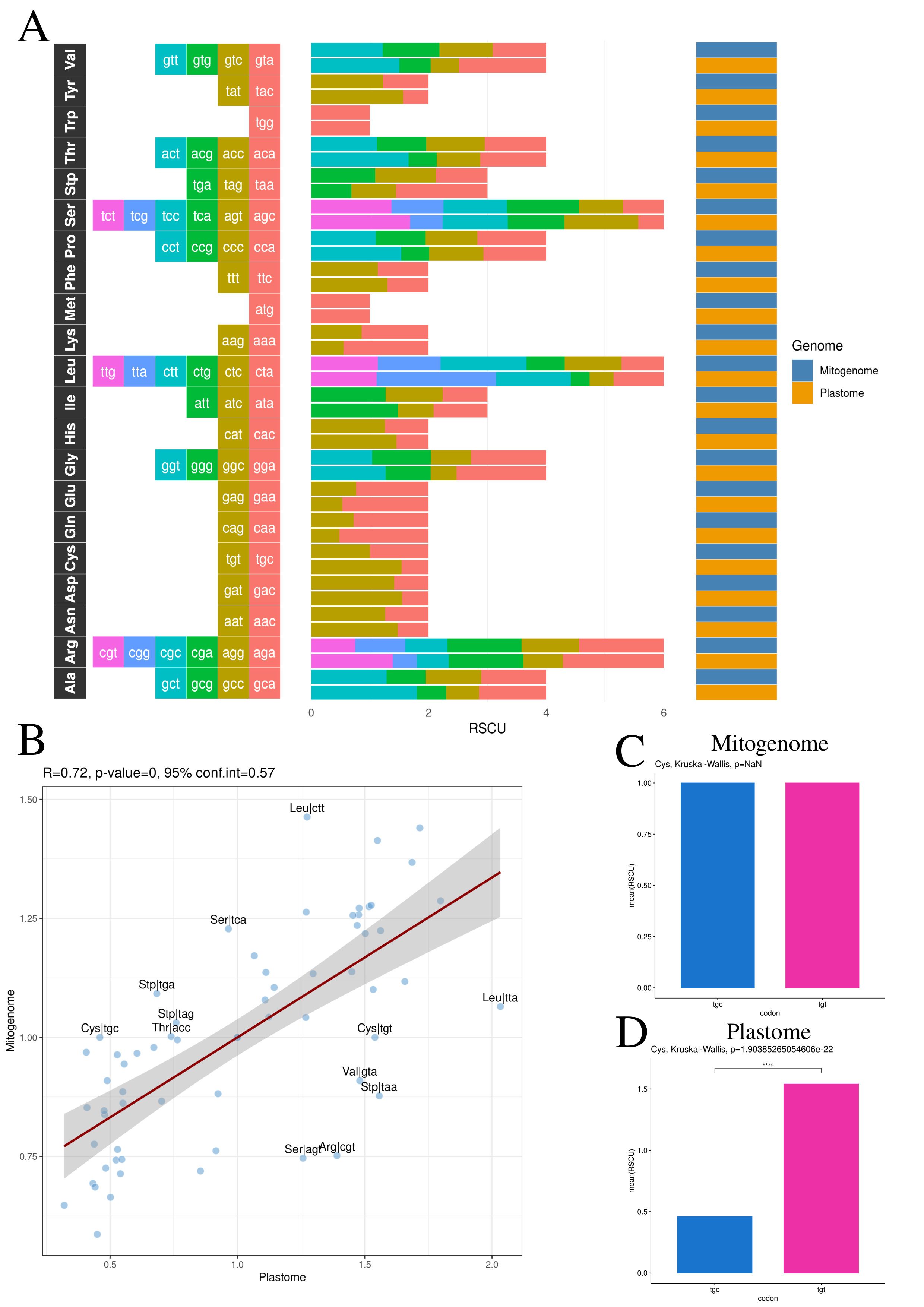

Supplement: Supplementary file 3 — Fig. S3. Codon usage in mitochondrial and plastid genome of Nassella tenuissima. [file CLA-41-358-s012.jpg]

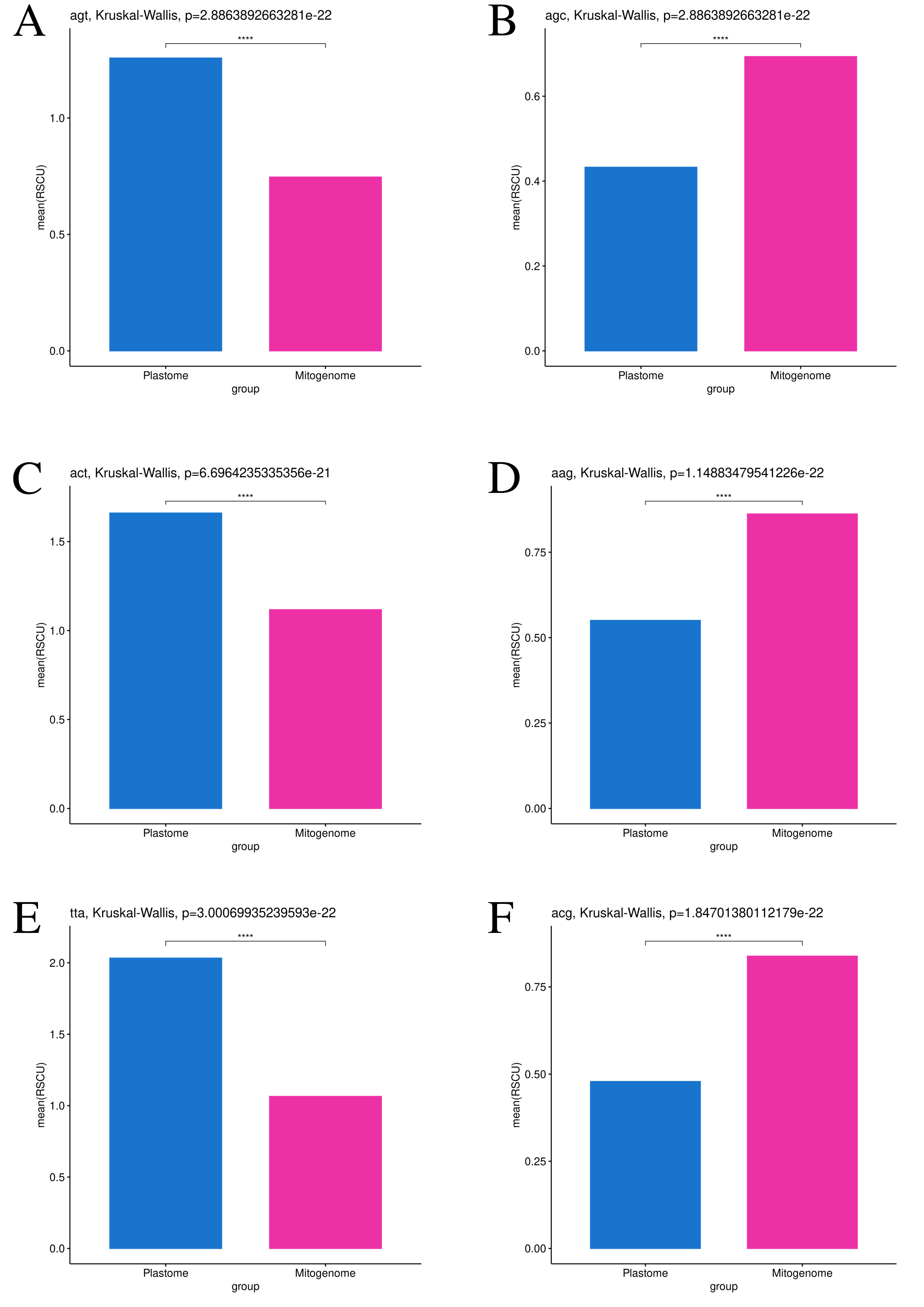

Supplement: Supplementary file 4 — Fig. S4. Statistically significant differences in codon usage frequencies between the plastomes and mitogenomes in Stipeae. [file CLA-41-358-s001.jpg]

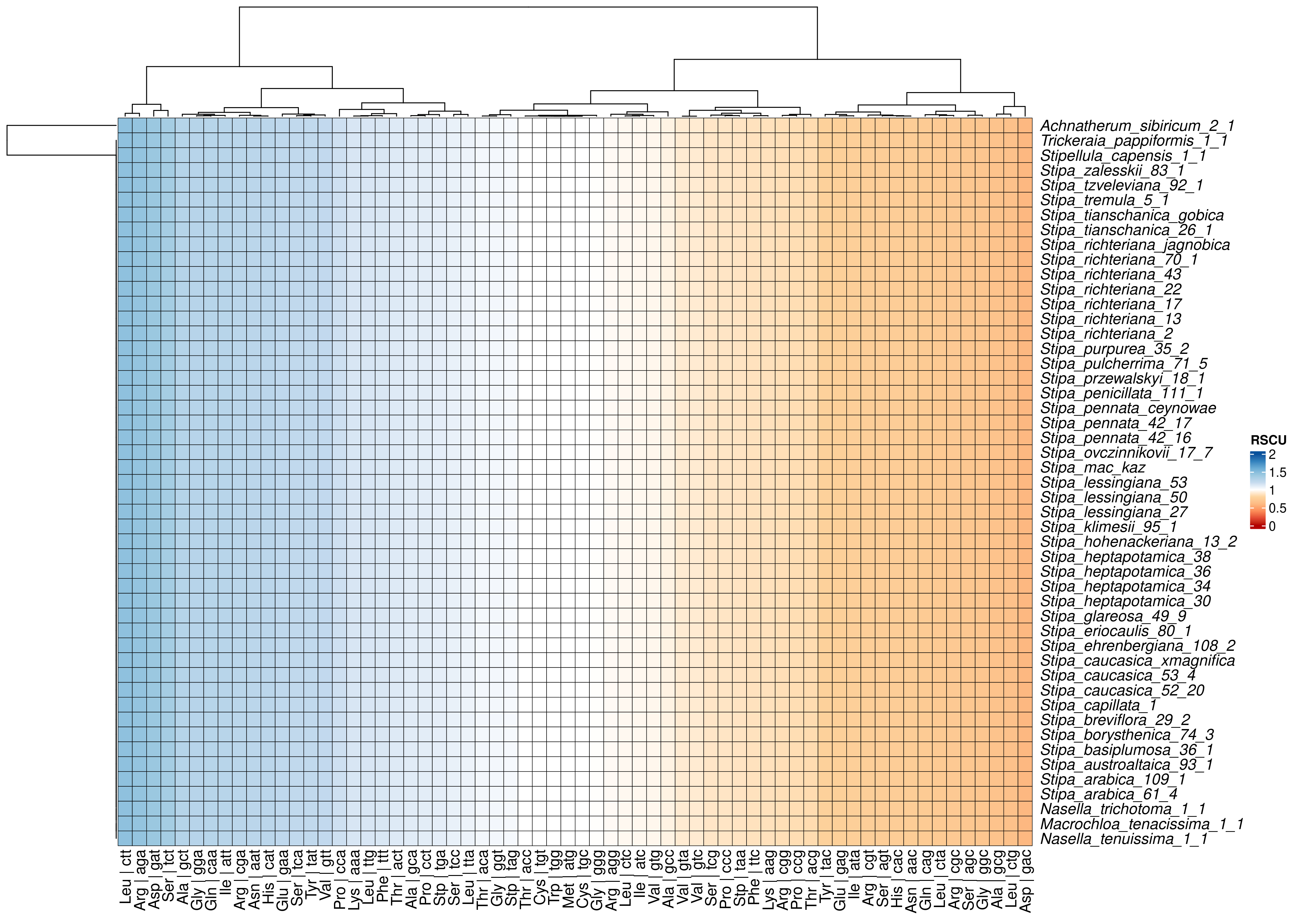

Supplement: Supplementary file 5 — Fig. S5. The RSCU heatmap of mitochondrial codons shows species preference of codon usage: dark orange and light orange colours represent less preferred codons, white colour represents codons that are neither less preferred nor more preferred, and light blue and dark blue colours represent more preferred codons. [file CLA-41-358-s007.png]

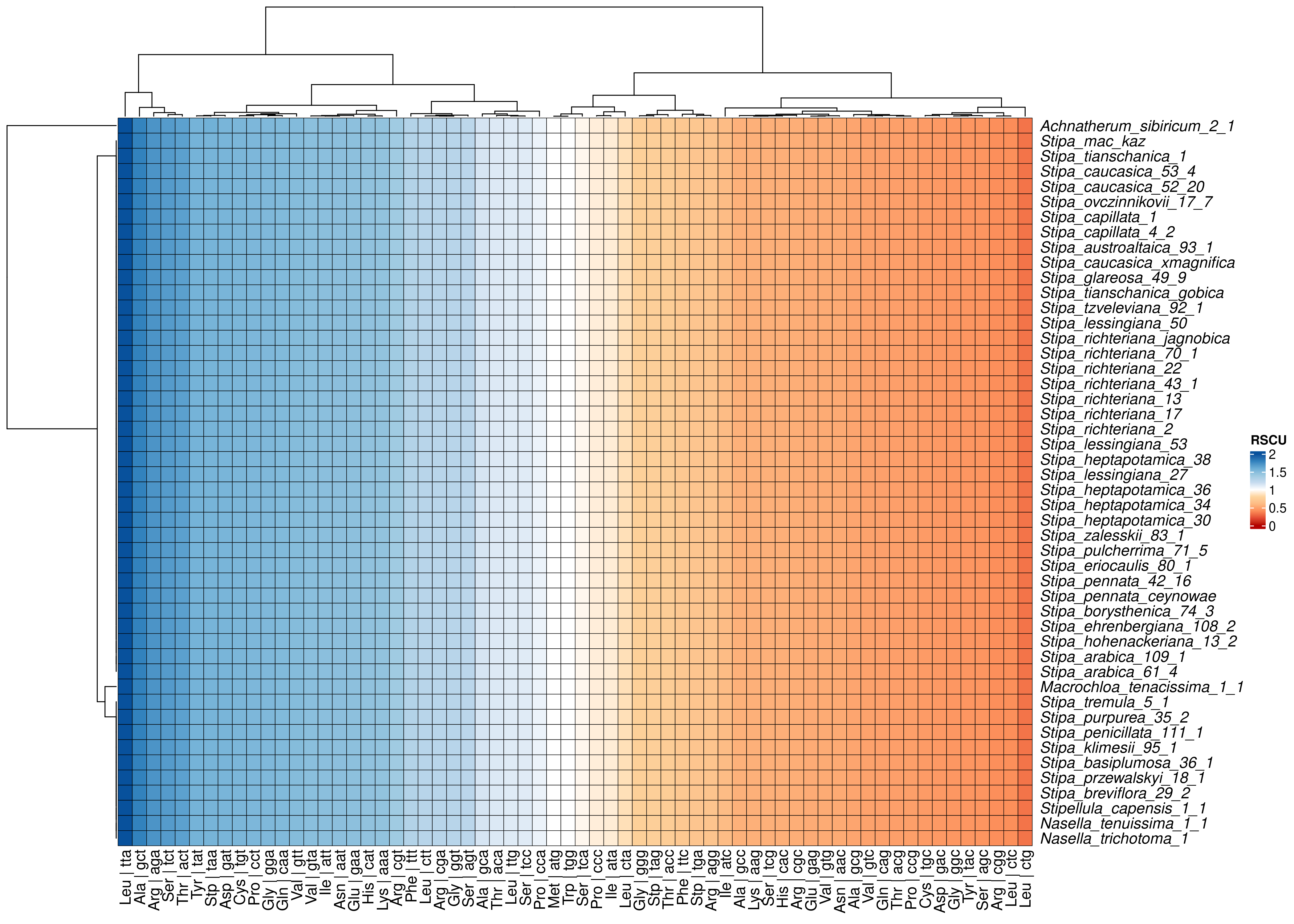

Supplement: Supplementary file 6 — Fig. S6. The RSCU heatmap of plastome codons shows species preference of codon usage: dark orange and light orange colours represent less preferred codons, white colour represents codons that are neither less preferred nor more preferred, and light blue and dark blue colours represent more preferred codons. [file CLA-41-358-s004.png]

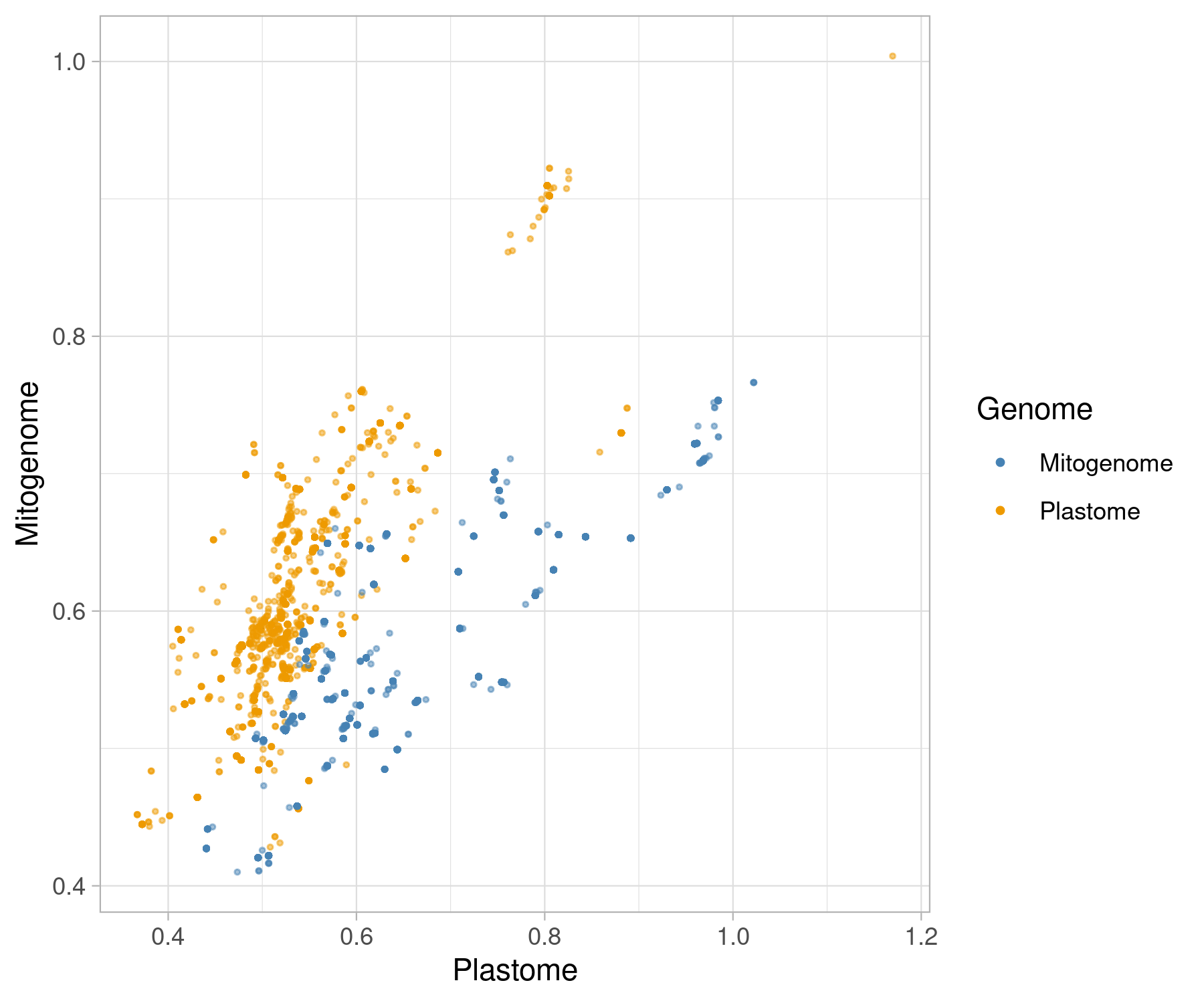

Supplement: Supplementary file 7 — Fig. S7. MILC analysis of codon usage bias between MT and PT genomes. [file CLA-41-358-s013.png]

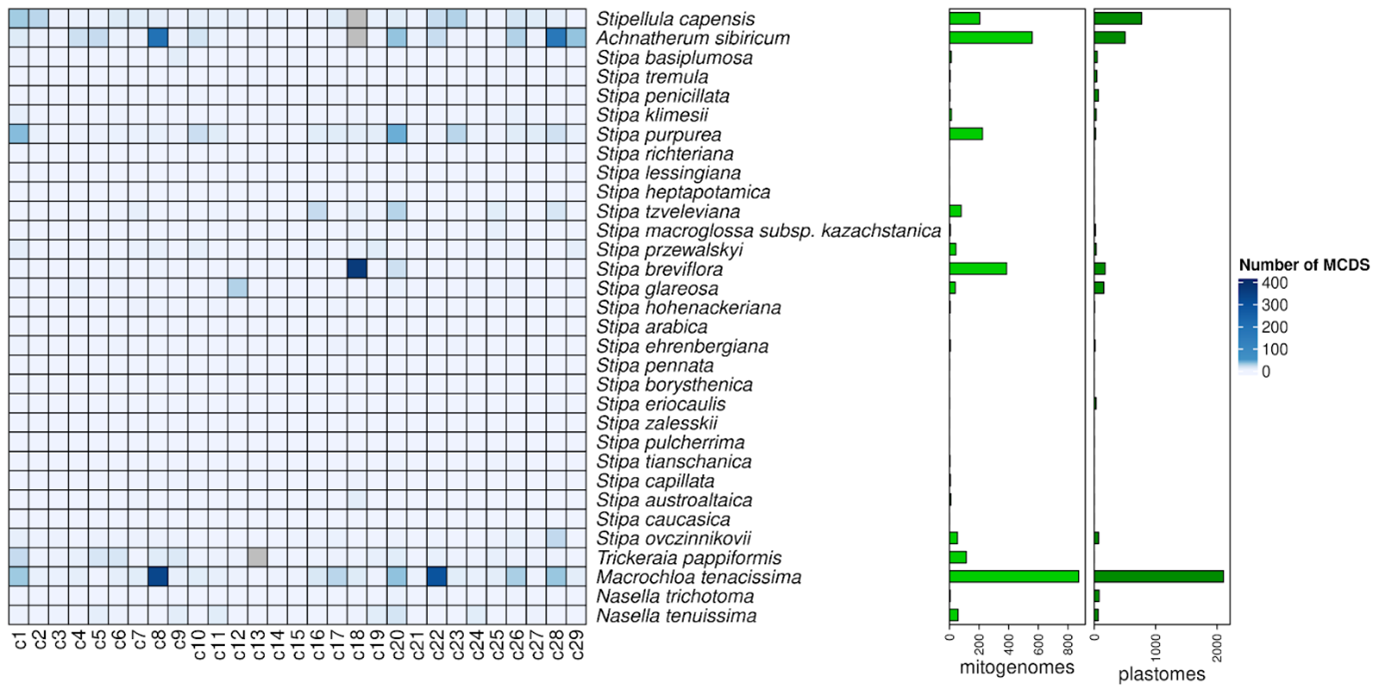

Supplement: Supplementary file 8 — Fig. S8. Comparison of the effectiveness of species identification using the analysed mitobarcodes (left plot) and using plastomes (excluding one IR) and 29 mitobarcodes (right plot). [file CLA-41-358-s015.png]

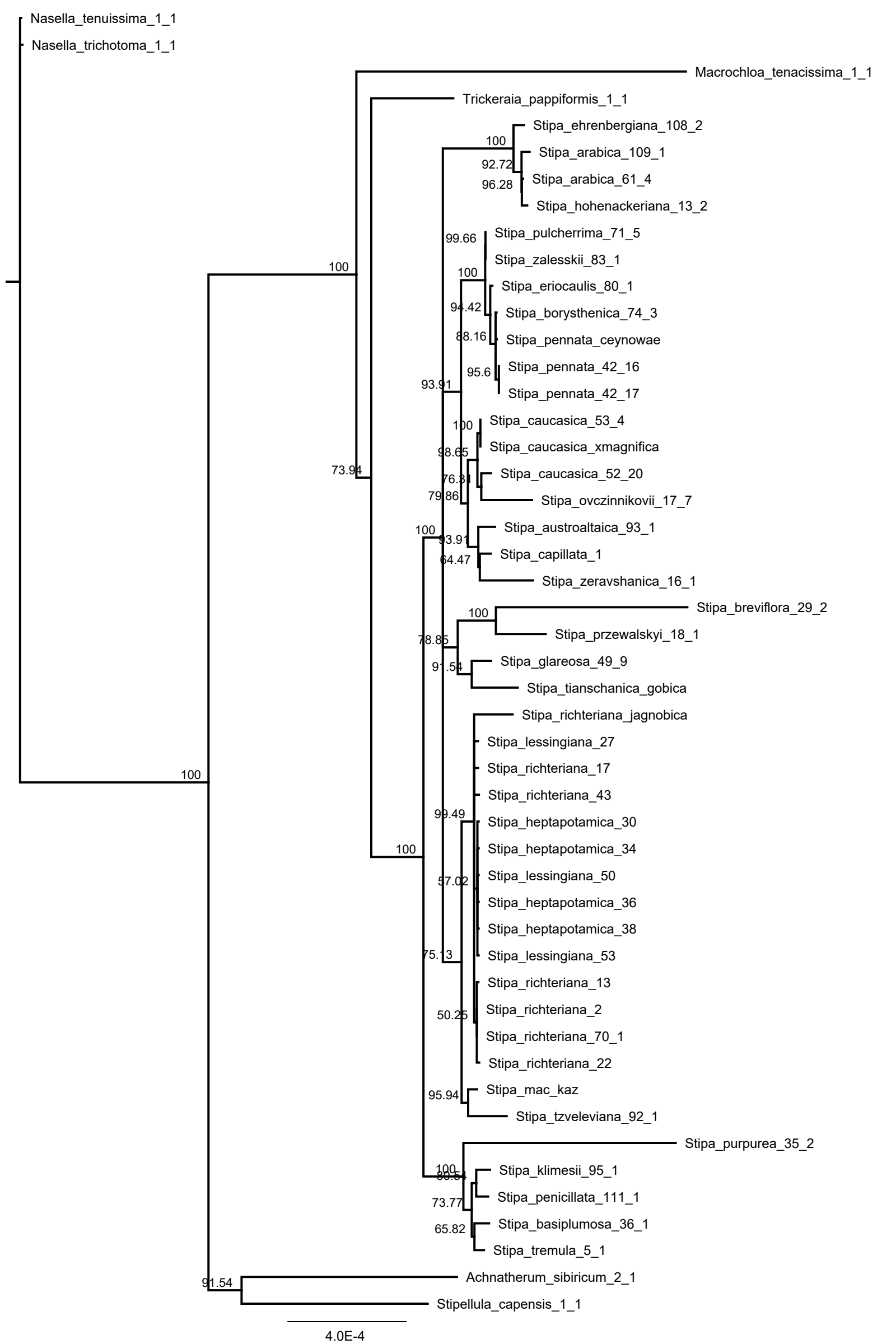

Supplement: Supplementary file 9 — Fig. S9. Evolutionary analysis based on mitochondrial fragments (c1–c29 described in the text). [file CLA-41-358-s002.pdf]
