# Supplementary material for: Phylogenetic reconstruction and species delimitation in Stipeae with special reference to Stipa (Poaceae, Pooideae) using mitochondrial genomes
Source: Cladistics. 2025 May 28;41(4):358–71. doi: 10.1111/cla.12618 (PMC12267929; doi:10.1111/cla.12618)
Supplement: Supplementary file 16 — Appendix S1. Characteristics of the mitochondrial genome of Nassella tenuissima. [file CLA-41-358-s006.docx]

**Characteristics of the mitochondrial genome of *Nassella tenuissima***

The assembled mitochondrial genome of *N. tenuissima* had the size of 424,370 bp the form of a single circular chromosome. Base composition of the genome was as follows: A 27.7%, C 22.2%, T 27.9%, G 27.9% with GC content of 44.4%.

Annotation of the mitogenome revealed the presence of 37 protein-coding genes (PCGs), including all the mitochondrial core-genes according to the classification of Skippington and coauthors (Skippington et al., 2015). The genes encode 19 proteins associated with the electron transport chain. These include nine subunits of complex I: NADH dehydrogenase subunits 1, 2, 3, 4, 4L, 5, 6, 7, and 9 (*nad*1, 2, 3, 4, 4L, 5, 6, 7, and 9); one subunit of complex III: apocytochrome b (*cob*); three subunits of complex IV: cytochrome c oxidase subunits *(cox*1, 2, and 3); and five subunits of complex V: ATP synthase F1 subunits (*atp*1, 4, 6, 8 (duplicated), and 9). The *nad*1 gene and *nad*5 gene consisted of three intervals. No genes were identified for encoding subunits of complex II: succinate dehydrogenase subunits 3 and 4 (*sdh*3 and *sdh*4). Additionally, four genes encode proteins involved in cytochrome c biogenesis, specifically subunits B, C, and F (*ccm*B, C, FC and FN). The *mat*R and *mtt*B genes encode maturase and transport membrane proteins, respectively. Finally, twelve genes *(rps*1, *rps*3, *rps*4, *rps*7, *rps*12 (duplicated), *rps*13, *rps*14, *rps*19, *rpl*2, *rpl*5 and *rpl*16) encode ribosomal proteins. In total, PCGs accounted for 12.96% of the mitogenome. Furthermore, the presence of 27 tRNA genes and six ribosomal RNA (rRNA) genes, namely two copies of *rrn*5, *rrn*18 and *rrn*26, was observed. Moreover 29 open reading frames (ORF) and one hypothetical protein were identified. The order and localization of these genes are illustrated in Fig. 1, Fig. S1 and reported in Table S2.

The mitochondrial structure and gene order of the *N. tenuissima* mitogenome is significantly different from the structure of the *Stipa capillata* mitogenome (Fig. 1). A comparative analysis of these two genomes revealed that their homologous fragments had a total length of 384,750 base pairs, representing 87.84% of the MZ161090, MZ161091, MZ161092, and MZ161093 genomes, and 303,838 base pairs on the homologous side of *N. tenuissima*, representing 71.60% of the entire *Nasella* genome. In detail, 107,357 base pairs in MZ161090 (93.02%) were homologous to 105,903 base pairs in *N. tenuissima* (24.96%), 39,311 base pairs in MZ161091 (73.68%) were homologous to 39,444 base pairs in *N. tenuissima* (9.3%), 56,441 base pairs in MZ161092 (84.43%) were homologous to 57,322 base pairs in *N. tenuissima* (13.51%), and 181,641 base pairs in MZ161093 (89.73%) were homologous to 181,169 base pairs in (42.69%).

The RSCU analysis (The Relative Synonymous Codon Usage) allowing to compare the frequency of codons used to encode individual amino acids between organellar genomes of *Nassella tenuissima*, showed diversity in codon usage between the plastid and mitochondrial genome (R=0.72) (Figure 6, Table S5). Cases have been observed where codons preferred in the mitogenome were not preferred in the plastome: Serine tca (RSCU_mt_=1.23; RSCU_pt_=0.96) and Stop codon tga (RSCU_mt_=1.09; RSCU_pt_=0.68) and tag (RSCU_mt_=1.03; RSCU_pt_=0.76). The opposite situation, *i.e.* the preference for using codons in the plastome and the reduction in the frequency of their use in the mitogenome, concerned four codons: Serine agt (RSCU_mt_=0.75; RSCU_pt_=1.26), Arginine cgt (RSCU_mt_=0.75; RSCU_pt_=1.39), Valine gta (RSCU_mt_=0.91; RSCU_pt_=1.48) and Stop codon taa (RSCU_mt_=0.88; RSCU_pt_=1.56). Cysteine ​​tgc (RSCU_mt_=1; RSCU_pt_=0.46) and Threonine acc (RSCU_mt_=1; RSCU_pt_=0.74) were unbiased in the mitogenome and unpreferred in the plastome. The highest frequency in both organellar genomes was shown by Leucine, however in mitogenome codon ctt (RSCU_mt_=1.46) was the most frequently used while in plastome it was the tta codon (RSCU_pt_=2.03). The RSCU analysis in case of several amino acids (Asn, Asp, Gln, Glu, His, Ile, Lys, Phe, Ser, The, Tyr) showed the preference of the same codon in mitogenome and plastome. Codon Cys tgt was unbiased in the mitogenome and preferred in the plastome. However, for cysteine ​​a clear preference of one codon (tgt) was observed in the plastid genome (RSCU_pt_=1.54), while in the mitogenome the frequency of both codons is unbiased (RSCU_mt_=1). The MILC method was used to quantify the difference in codon usage distance between the plastome and the mitochondrial genomes. A separation of mitochondrial and plastid genes was observed (Fig. S5). Significant changes in codon usage preferences between the organellar genomes were observed in six codons: aag, acg, act, agc, agt and tta (Fig. S6). Analysis of codon usage in Stipeae showed that it is phylogenetically conserved in both the plastome and the mitogenome (Fig. S7, Fig. S8)

**Reference**

Skippington, E., Barkmanb, T.J., Ricea, D.W., Palmera, J.D. 2015. Miniaturized mitogenome of the parasitic plant viscum scurruloideum is extremely divergent and dynamic and has lost all nad genes. Proceedings of the National Academy of Sciences of the United States of America. 112, E3515–E3524. doi:10.1073/pnas.1504491112


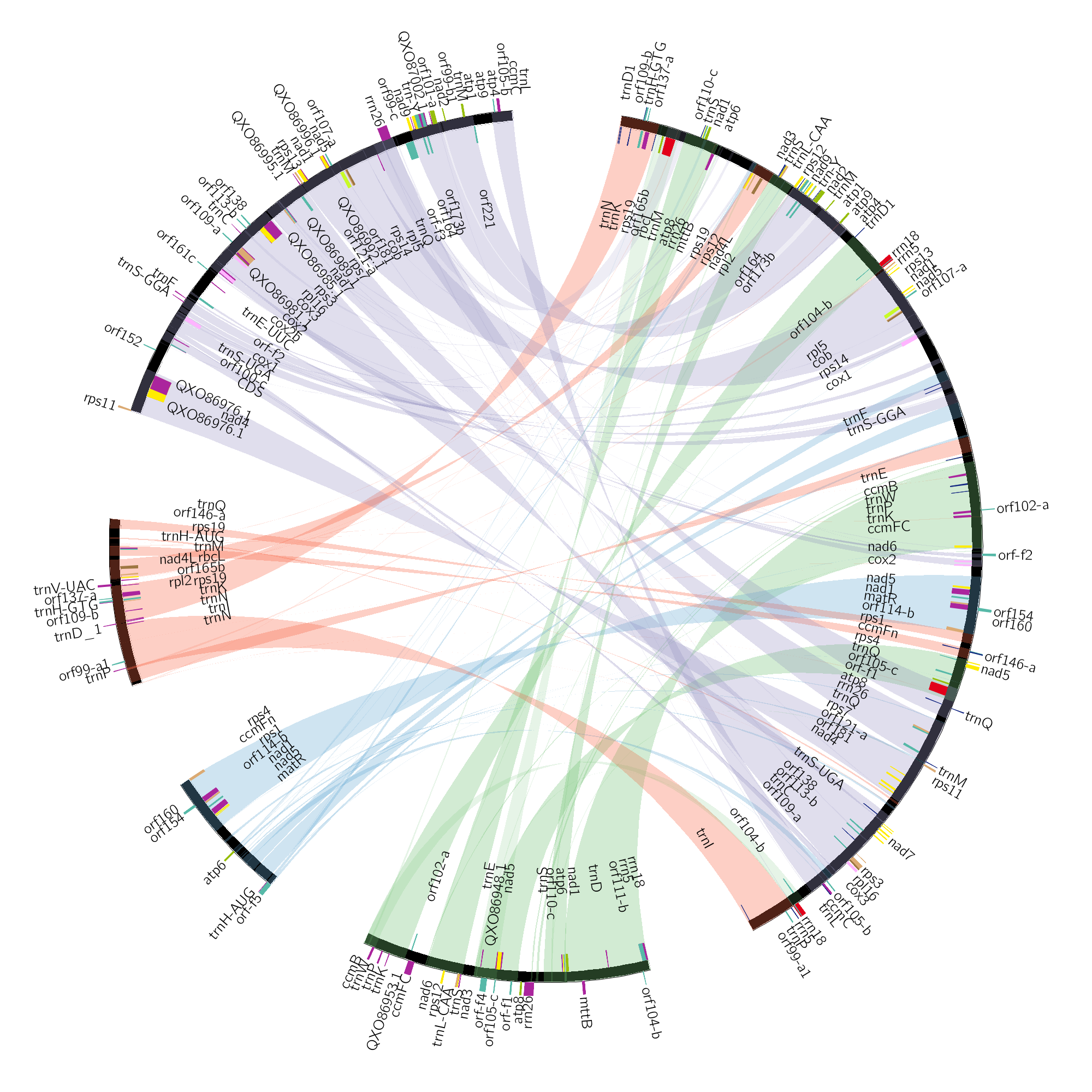


**Fig. S2**

Comparison of four mitochondrial contigs of *Stipa capillata* (left) and *Nassella tenuissima* (right) mitochondrial genome structure and gene order. Genes inside and outside the circle are transcribed in counterclockwise and clockwise directions, respectively. Ribbons connect highly homologous sequences between the analysed species. Each of the contigs of *S. capillata* was marked with a different colour: c1: green, c2: blue, c3: purple, c4: orange.

**
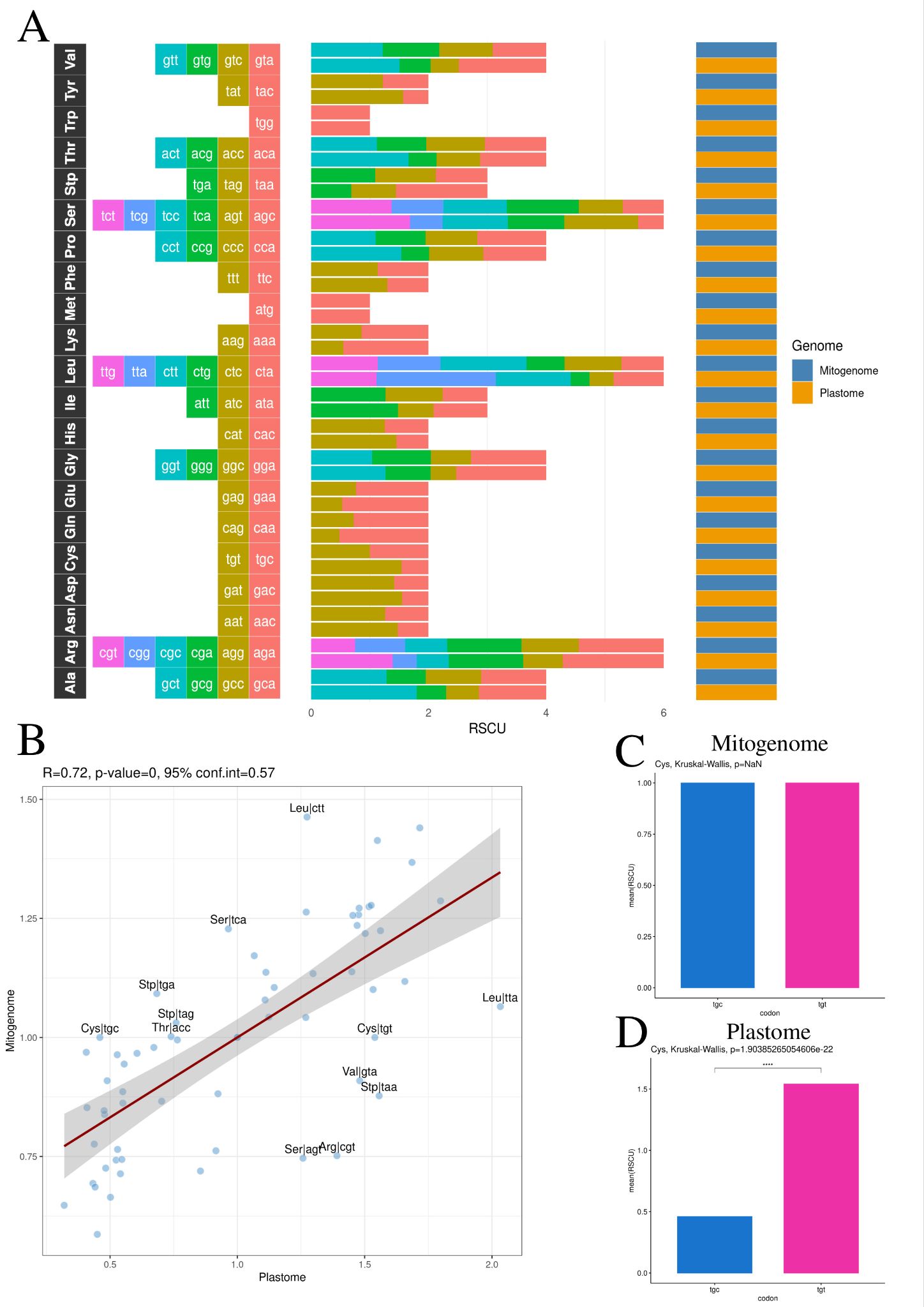
**

**Fig. S3**

Codon usage in mitochondrial and plastid genome of *Nassella tenuissima*.

**A.** The Relative Synonymous Codon Usage (RSCU) for each triplet coding amino-acid. **B.** Correlation plot of CU in plastome and mitogenome. The red line represents the Pearson correlation coefficient value. The grey bands around the line represent the standard error of the regression line. **C-D.** Comparison of RSCU for Cysteine between mitochondrial (C) and plastome (D) genomes of *Nassella tenuissima*. Asterisks indicate the credibility level p < 0.001.
