# Supplementary material for: Phylogenetic reconstruction and species delimitation in Stipeae with special reference to Stipa (Poaceae, Pooideae) using mitochondrial genomes
Source: Cladistics. 2025 May 28;41(4):358–71. doi: 10.1111/cla.12618 (PMC12267929; doi:10.1111/cla.12618)
Supplement: Supplementary file 10 — Fig. S10. Plastome‐based evolutionary analysis by Maximum Likelihood method based on the K3Pu + F + I model of nucleotide substitution. [file CLA-41-358-s016.pdf]

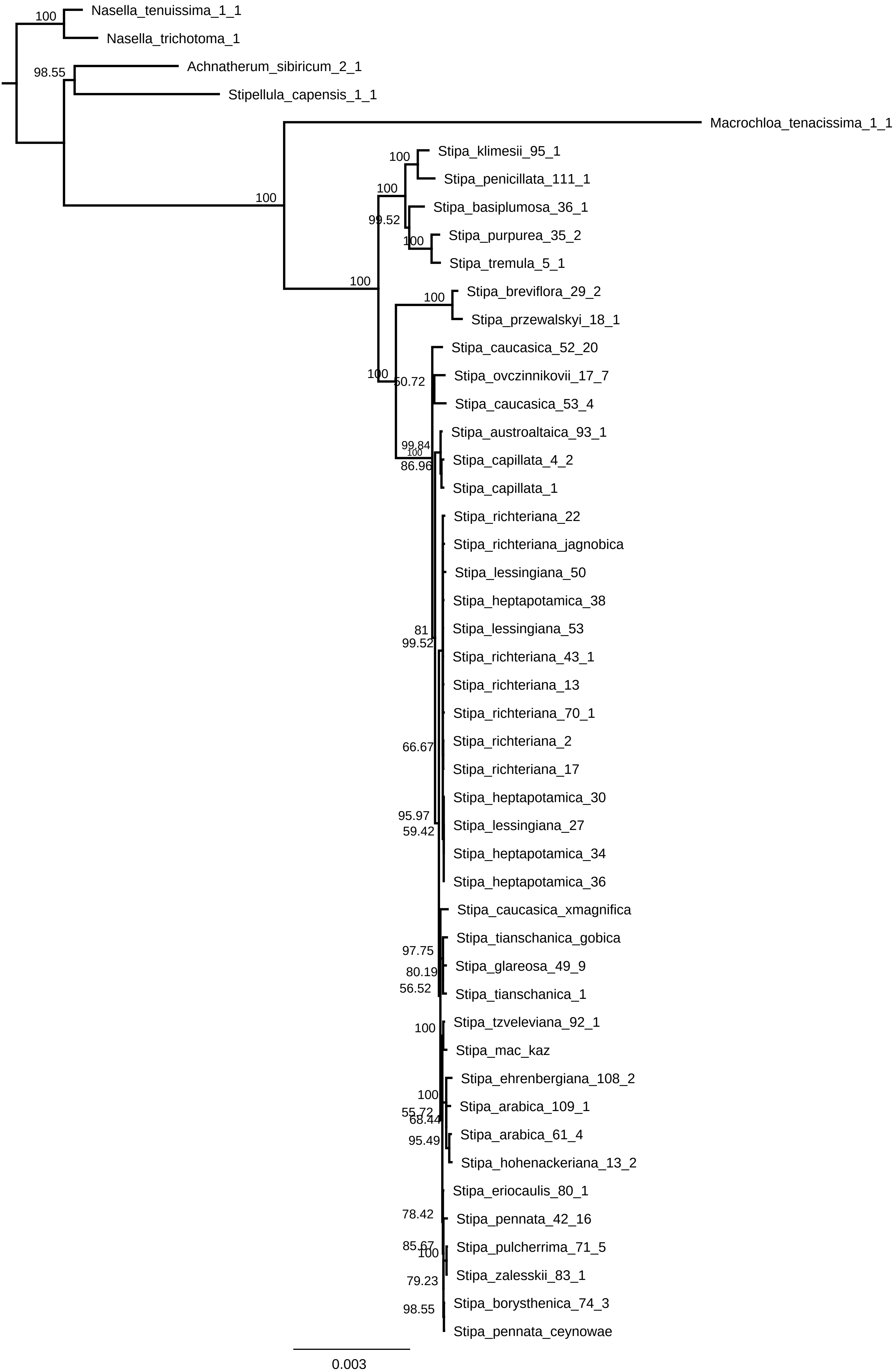
Nasella_tenuissima_1_1
100
Nasella_trichotoma_1
Achnatherum_sibiricum_2_1
98.55
Stipellula_capensis_1_1
Macrochloa_tenacissima_1_1
Stipa_klimesii_95_1
100
Stipa_penicillata_111_1
100
100
Stipa_basiplumosa_36_1
99.52
Stipa_purpurea_35_2
100
Stipa_tremula_5_1
100
Stipa_breviflora_29_2
100
Stipa_przewalskyi_18_1
Stipa_caucasica_52_20
Stipa_ovczinnikovii_17_7
100
50.72
Stipa_caucasica_53_4
Stipa_austroaltaica_93_1
99.84
100
Stipa_capillata_4_2
86.96
Stipa_capillata_1
Stipa_richteriana_22
Stipa_richteriana_jagnobica
Stipa_lessingiana_50
Stipa_heptapotamica_38
81
Stipa_lessingiana_53
99.52
Stipa_richteriana_43_1
Stipa_richteriana_13
Stipa_richteriana_70_1
Stipa_richteriana_2
66.67
Stipa_richteriana_17
Stipa_heptapotamica_30
95.97
Stipa_lessingiana_27
59.42
Stipa_heptapotamica_34
Stipa_heptapotamica_36
Stipa_caucasica_xmagnifica
Stipa_tianschanica_gobica
97.75
Stipa_glareosa_49_9
80.19
56.52
Stipa_tianschanica_1
Stipa_tzveleviana_92_1
100
Stipa_mac_kaz
Stipa_ehrenbergiana_108_2
100
Stipa_arabica_109_1
55.72
68.44
Stipa_arabica_61_4
95.49
Stipa_hohenackeriana_13_2
Stipa_eriocaulis_80_1
78.42
Stipa_pennata_42_16
Stipa_pulcherrima_71_5
85.67
100
Stipa_zalesskii_83_1
79.23
Stipa_borysthenica_74_3
98.55
Stipa_pennata_ceynowae
0.003
